# A comprehensive study of the promoting effect of manganese on white rot fungal treatment for enzymatic hydrolysis of woody and grass lignocellulose

**DOI:** 10.1186/s13068-021-02024-7

**Published:** 2021-09-06

**Authors:** Xiao Fu, Jialong Zhang, Xiangyu Gu, Hongbo Yu, Shulin Chen

**Affiliations:** 1 Key Laboratory of Molecular Biophysics of MOE, College of Life Science and Technology, Huazhong University of Science and Technology, Wuhan, 430074 China; 2 Department of Biological Systems Engineering, Washington State University, Pullman, WA 99164 USA

**Keywords:** Mn addition, Fungal treatment, Enzymatic hydrolysis, Delignification, β-O-4 cleavage, Principal component analysis

## Abstract

**Background:**

The efficiency of biological systems as an option for pretreating lignocellulosic biomass has to be improved to make the process practical. Fungal treatment with manganese (Mn) addition for improving lignocellulosic biomass fractionation and enzyme accessibility were investigated in this study. The broad-spectrum effect was tested on two different types of feedstocks with three fungal species. Since the physicochemical and structural properties of biomass were the main changes caused by fungal degradation, detailed characterization of biomass structural features was conducted to understand the mechanism of Mn-enhanced biomass saccharification.

**Results:**

The glucose yields of fungal-treated poplar and wheat straw increased by 2.97- and 5.71-fold, respectively, after Mn addition. Particularly, over 90% of glucose yield was achieved in Mn-assisted *Pleurotus ostreatus*-treated wheat straw. A comparison study using pyrolysis gas chromatography mass spectrometry (Py-GC/MS) and two-dimensional ^1^H–^13^C heteronuclear single quantum coherence (2D HSQC) nuclear magnetic resonance (NMR) spectroscopy was conducted to elucidate the role of Mn addition on fungal disruption of the cross-linked structure of whole plant cell wall. The increased C_α_-oxidized products was consistent with the enhanced cleavage of the major β-O-4 ether linkages in poplar and wheat straw lignin or in the wheat straw lignin–carbohydrate complexes (LCCs), which led to the reduced condensation degree in lignin and decreased lignin content in Mn-assisted fungal-treated biomass. The correlation analysis and principal component analysis (PCA) further demonstrated that Mn addition to fungal treatment enhanced bond cleavage in lignin, especially the β-O-4 ether linkage cleavage played the dominant role in removing the biomass recalcitrance and contributing to the glucose yield enhancement. Meanwhile, enhanced deconstruction of LCCs was important in reducing wheat straw recalcitrance. The findings provided not only mechanistic insights into the Mn-enhanced biomass digestibility by fungus, but also a strategy for improving biological pretreatment efficiency of lignocellulose.

**Conclusion:**

The mechanism of enhanced saccharification of biomass by Mn-assisted fungal treatment mainly through C_α_-oxidative cleavage of β-O-4 ether linkages further led to the decreased condensation degree in lignin, as a result, biomass recalcitrance was significantly reduced by Mn addition.

**Graphic abstract:**

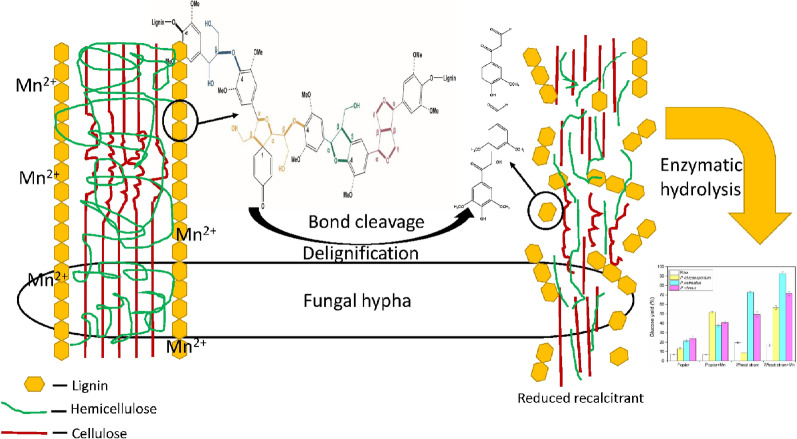

**Supplementary Information:**

The online version contains supplementary material available at 10.1186/s13068-021-02024-7.

## Background

Lignocellulosic feedstocks, such as poplar and wheat straw, have captured great attention as an abundant and renewable resource for producing biofuels and biochemicals [1]. The presence of lignin, hemicellulose and cellulose, and the linkages between those compositions provides chemical and physical recalcitrance to inhibit accessibility of cellulosic enzymes to the polysaccharides of the biomass to release sugar [2]. Pretreatment is therefore crucial to removing recalcitrance and enhancing biological conversion of lignocellulosic biomass to monosaccharides and other valuable chemicals [3]. In the natural ecosystem, some special species of white rot fungi were found selectively degrading lignin while keeping most of the polysaccharides in situ, which have been considered as the candidates to build a simple and environmentally friendly treatment method for biofuel production [4]. Due to the component heterogeneity and structure complexity of lignocellulose, only a few number of fungal–substrate combinations have been investigated promising for improving substrate saccharification [4]. Therefore, it is very important to develop a method that has the broad-spectrum effect on increasing efficiency of fungal treatment for different types of biomass. However, from industrial and economic perspective, the process of fungal treatment needs to be optimized to improve its efficiency to meet the requirement of biorefinery [5]. To improve the efficiency of fungal treatment, a number of approaches have been investigated [6–10]. Previous work showed that white rot fungi with Mn addition effectively increased biomass digestibility [9, 11, 12]. Song et al*.* reported that the Mn addition significantly enhanced lignin degradation and increased 50% of the glucose yield during *Irpex lacteus* treatment of corn stover [9]. According to these studies, Mn addition during fungal treatment is an easy way that has shown promising effect on enhancing saccharification of different types of lignocellulosic biomass. It is well known that Mn addition could regulate cellulolytic and ligninolytic enzymes activity during fungal treatment [9, 13–15]. Our recent work showed that Mn addition inhibited the expression of ligninolytic enzymes during *P. ostreatus* treatment. Instead, Mn addition regulated the synergistic network of Class II lignin-degrading peroxidases and H_2_O_2_ generation enzymes to enhance lignin degradation [16]. However, the effect of Mn addition on biomass structural changes, and the relationship between biomass structural changes and enhanced saccharification is still under the water. Moreover, it is not clear that whether the biomass structural changes caused by Mn addition are universal under different fungal treatments on various types of biomass.

The plant cell wall of lignocellulosic biomass is naturally resistant to cellulolytic enzyme hydrolysis due to compositional and structural factors, such as high lignin and hemicellulose contents, and various types of lignin linkages bridged to lignocellulosic matrix [17]. Previous work has shown that the surface lignin plays a significant role in lowering the enzymatic hydrolysis of biomass, due to its physically impeding or nonspecifically adsorbing cellulases [18]. In general, the major recalcitrance derives from lignin, which consists of three phenylpropane units: guaiacyl unit (G), syringyl unit (S), and *p*-hydroxyphenyl unit (H) polymerized through a serious of linkages with disordered repeat units [19]. The β-O-4 linkage is the most abundant inter-unit linkages in poplar (60–70%) and wheat straw (75%) and it is also most easily degraded during fungal treatment [20, 21]. Other inter-unit linkages such as carbon–carbon bonds including β-5, β-1 and 5–5, are more resistant to fungal degradation [8]. Besides lignin, lignin–carbohydrate complexes (LCCs) are reported be acted as shield protecting cellulose from fungal attacking [22]. Therefore, lignin, hemicellulose and linkages between these compositions are implicated in biomass to build a strong recalcitrance toward fungal attack. Since substrates differ in composition and enzymes differ in fungi species, previous studies mainly focused on choosing a specific fungus–substrate combination for obtaining the best yield of saccharification [23–25]. Meanwhile, comparing efficiency difference between different Mn-assisted fungal treatments is difficult because of the variation in fungus species, biomass types, culture conditions and analytical methods. Consequently, there has not been a study about the broad-spectrum mechanism how Mn addition improves enzymatic saccharification.

This study was therefore conducted to fill the knowledge gap. Three species of fungi were *Phanerochaete chrysosporium*, *Pleurotus ostreatus* and *Physisporinus vitreus* and the biomass included wheat straw as grass type and poplar as woody biomass. Py-GC/MS and HSQC NMR were used to investigate biomass chemical and structural changes. Correlation analysis and principal component analysis (PCA) were performed to identify the key factors affecting the biomass digestibility and the chemical and structural changes of the treated biomass were analyzed [26, 27]. Furthermore, the following statistical analysis was conducted to provide us the relationship between biomass features and subsequent enzymatic hydrolysis. The overall results of the study were expected to give us a better understanding of the mechanism on enhanced fungal biomass digestibility by Mn addition at structural level. The comparison of biomass linkages and structural features under various conditions (with and without Mn addition) provide in-depth information of fungal modification and degradation of biomass components especially for lignin degradation, enable expanding the knowledge base towards guiding an efficient fungal treatment strategy for saccharification.

## Results and discussion

### Impact of Mn addition on enzymatic hydrolysis

**Fig. 1:**
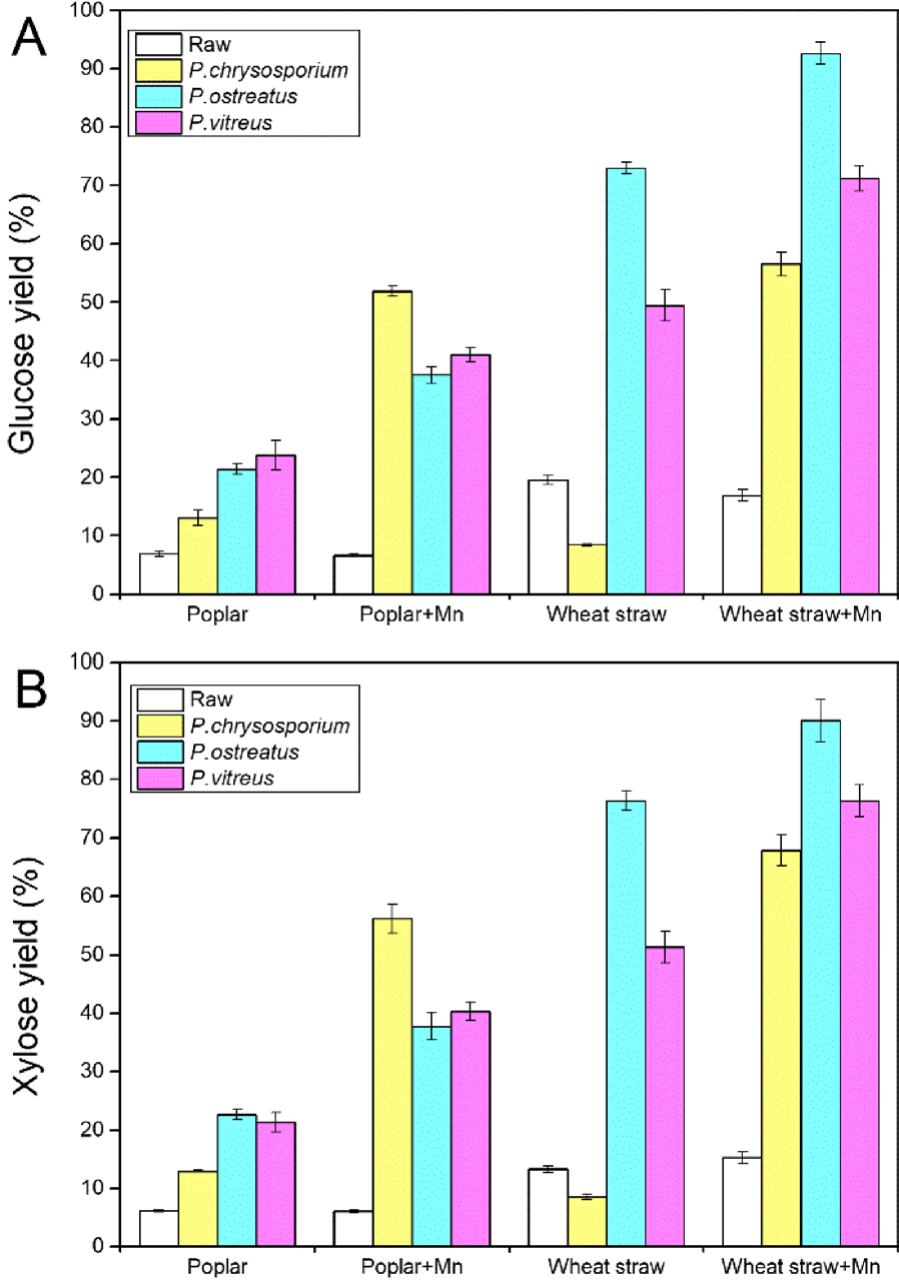
Glucose (**A**) and xylose (**B**) yields of poplar and wheat straw for different fungal treatments

Accessibility of cellulose to cellulosic enzymes was significantly enhanced by Mn addition during fungal treatment for both poplar and wheat straw (Fig. 1A). The cellulose conversions increasing from 13.05, 21.42 and 23.83% in *P. chrysosporium* (*Pc*), *P. ostreatus* (*Po*) and *P. vitreus* (*Pv*) treated poplar up to 51.87, 37.57 and 40.99% in Mn-assisted *P. chrysosporium* (*PcM*), *P. ostreatus* (*PoM*) and *P. vitreus* (*PvM*) treatment, separately. Digestibility of wheat straw celluloses increased from 8.43, 72.98 and 49.44% to 56.57, 92.63 and 71.20% after Mn addition to *Pc*, *Po* and *Pv* treatment, separately. For fungal-treated biomass, the digestibility of hemicellulose has the similar level to cellulose conversion (Fig. 1B). Mn addition without fungal cultivation to biomass has tiny effects on the cellulose and hemicellulose conversion (Fig. 1A, B). In this study, the highest cellulose conversion reached to 92.63% was obtained in 28 days *PoM*-treated wheat straw, which is comparative to current leading physical and/or thermochemical treatments [28], suggesting that Mn-assisted fungal treatment has a great potential to be an alternative to the existing lignocellulose pretreatment approaches, such as thermo-chemical or chemi-mechanical treatments.

### Impact of Mn addition on chemical composition of treated biomass

Impact of Mn addition on chemical compositions (lignin, hemicellulose, and cellulose) of the treated biomass is shown in Table 1. The contents of lignin (24.66, 25.72, 25.04% and 19.35, 14.25, 14.57%), hemicellulose (18.40, 19.05, 19.40% and 22.46, 19.52 and 20.36%) and cellulose (46.08, 52.65, 53.35% and 38.61, 42.04, 41.70%) were detected in *P. chrysosporium* (*Pc*), *P. ostreatus* (*Po*) and *P. vitreus* (*Pv*) treated poplar and wheat straw, respectively. The lignin and hemicellulose contents in fungal-treated biomass were lower than in the raw poplar (27.19 and 18.34%) and wheat straw (19.35 and 22.16%), whereas cellulose contents were higher than in the raw poplar (47.19%) and wheat straw (39.82%), respectively. The total contents of lignin, hemicellulose and cellulose in fungal-treated samples were higher than in the raw biomass, probably due to that the ethanol–benzene extractives such as protein or lipids in the biomass was degraded by fungus (lignin + hemicellulose + cellulose + extractives = 100%). It is shown that Mn addition not only enhanced lignin and hemicellulose degradation, but also inhibited cellulose consumption during fungal treatments. In all treated samples, the minimum contents of lignin (20.30, 21.54, 21.12% and 16.29, 11.95, 12.01%) and hemicellulose (18.40, 17.27, 17.83% and 21.75, 16.95, 18.66%) were observed in Mn-assisted *P. chrysosporium* (*PcM*), *P. ostreatus* (*PoM*) and *P. vitreus* (*PvM*)-treated poplar and wheat straw, respectively. Meanwhile, the maximum contents of cellulose (54.44, 55.30, 56.67% and 44.66, 43.24, 42.15%) were detected in *PcM*, *PoM* and *PvM*-treated poplar and wheat straw, respectively. In the plant cell wall, lignin is associated with carbohydrates (mainly hemicellulose) via LCC ester and ether bonds [29]. Additionally, a thin layer of lignin and/or hemicellulose rich residue is coated on the elementary microfibrils which consisted of cellulose–hemicellulose within lignocellulosic plant cell walls. As a result, hemicellulose loss cannot be avoided along with lignin removal during fungal treatment [30]. The specific degrading of hemicellulose was found in line with lignin removal during fungal treatment of lignocellulosic biomass [8, 25, 30]. The enhanced degradation of lignin and hemicellulose by Mn addition is probably directly linked. During fungal treatment, selective delignification was the major factor caused by Mn addition which further enhanced the enzymatic hydrolysis [9, 11]. Usually, the remaining cellulose after fungal treatment was more accessible for enzymes than before treatment [31]. Meanwhile, there was more cellulose mass fraction retained in Mn-assisted fungal-treated biomass. Thus, more cellulose could be easily converted to glucose in Mn-assisted fungal-treated biomass. This is consistent with the increasing of glucose yields in treated biomass with Mn addition (Fig. 1A). The highest cellulose conversion was 568.57 g/kg glucose from biomass which was obtained in 28 days *PoM*-treated wheat straw, could be comparative to current leading treatments (196.0 to 442.5 g/kg glucose from biomass) [28]. In addition, the weight loss of poplar and wheat straw were also increased by Mn addition during fungal treatments (Additional file 1: Figure S1).

**Table 1:** Chemical compositions of lignocellulosic biomass untreated and treated by *P. chrysosporium*, *P. ostreatus* and *P. vitreus*

Treatment	Poplar					Wheat straw				
	Lignin (%)	Cellulose (%)	HE^c^ (%)	Ash (%)	Extractive (%)	Lignin (%)	Cellulose (%)	HE^c^ (%)	Ash (%)	Extractive (%)
Raw^a^	27.19 ± 0.66	47.19 ± 0.25	18.34 ± 0.67	0.96 ± 0.32	6.32 ± 1.17	19.35 ± 0.68	39.82 ± 0.92	22.16 ± 0.11	4.20 ± 0.53	14.47 ± 2.88
RawM^b^	28.01 ± 0.76	45.32 ± 2.36	18.34 ± 0.37	1.32 ± 0.12	7.01 ± 0.77	19.63 ± 0.25	40.20 ± 0.72	23.15 ± 0.21	4.85 ± 0.53	12.17 ± 2.88
*Pc*	24.66 ± 0.40	46.08 ± 1.26	18.04 ± 0.32	0.03 ± 0	10.83 ± 1.23	19.35 ± 0.10	38.61 ± 0.39	22.46 ± 0.27	4.53 ± 0.85	15.05 ± 1.35
*Po*	25.72 ± 0.47	52.65 ± 1.04	19.05 ± 0.76	0 ± 0	2.58 ± 0.53	14.25 ± 0.42	42.04 ± 0.95	19.52 ± 0.49	4.05 ± 0.92	20.14 ± 2.51
*Pv*	25.09 ± 0.77	53.35 ± 1.15	19.40 ± 0.71	0.41 ± 0.03	1.75 ± 0.36	14.57 ± 0.96	41.70 ± 0.23	20.36 ± 1.11	3.38 ± 0.82	19.99 ± 2.69
*PcM*	20.30 ± 0.11	54.44 ± 0.94	17.04 ± 0.94	0.11 ± 0.01	8.12 ± 1.36	16.29 ± 0.06	44.66 ± 0.13	21.75 ± 0.41	5.35 ± 0.57	11.94 ± 1.25
*PoM*	21.54 ± 1.17	55.30 ± 0.97	17.28 ± 0.54	0.17 ± 0.02	5.71 ± 1.03	11.95 ± 0.94	43.24 ± 0.57	16.94 ± 0.26	5.26 ± 0.37	22.61 ± 2.31
*PvM*	21.12 ± 0.91	56.67 ± 1.13	17.83 ± 1.21	0.46 ± 0.08	3.92 ± 0.95	12.01 ± 0.71	42.15 ± 0.98	18.66 ± 0.76	4.51 ± 0.92	22.67 ± 3.64

*Pc P. chrysosporium, Po P. ostreatus, Pv P. vitreus, PcM P. chrysosporium* + Mn, *PoM P. ostreatus* + Mn*,* and *Pv P. vitreus* + Mn-treated lignocellulosic biomass

^a^Untreated lignocellulosic biomass

^b^Untreated lignocellulosic biomass with Mn addition

^c^Hemicellulose

### Structural features of fungal-treated lignocellulose

**Fig. 2:**
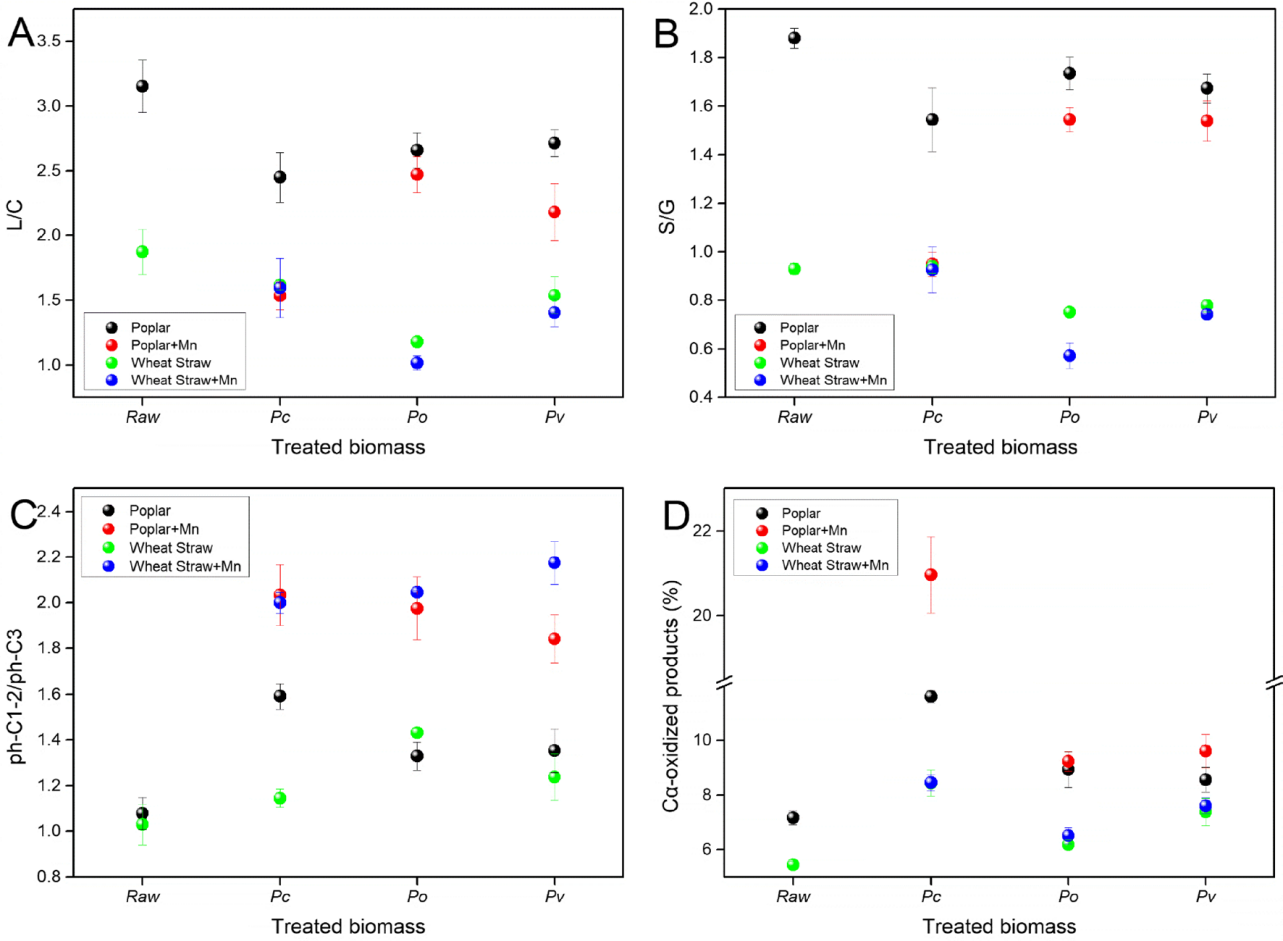
Main lignin features of poplar and wheat straw identified in Py-GC/MS

The lignin/carbohydrate (L/C) ratio of all treated samples slightly decreased. Samples with Mn addition decreased more in this ratio (Fig. 2A). This suggested that Mn addition assisted those fungal strains to preferentially degrade the lignin moiety and relatively enriched cellulose as a result of the treatment. The higher biodegradability of lignin compared with cellulose was also reported in other studies showing the decrease of the L/C ratio in fungal-treated biomass [31, 32]. Moreover, the Py-GC/MS analysis results showed the S/G ratio of lignin decreased after fungal treatment. Such decrease was also enhanced by Mn addition (Fig. 2B). In addition, several lignin S units (peak 33 in treated poplar and peak 26, 29, 30, 33 and 34 in treated wheat straw) could not be detected after fungal treatment (Additional file 1: Table S1 and S2). Consequently, less lignin S units were found in both the fungal-treated poplar and wheat straw. This result confirms the previous observation showing the decreased S/G ratio during fungal degradation of lignocellulosic biomass [31, 33, 34]. The result also suggests the higher biodegradability of the S units compared with G units. The preferential S units degradation by fungi was probably due to that, compared to G units S units include a relative low percentage of C–C bonds, higher degree of methoxylation, leading to a higher predominance of β-O-4 linkages. The β-O-4 linkages were less recalcitrant to fungal attack [30, 31, 35]. Decreased S/G ratio in the Mn-assisted biomass suggests that Mn addition during fungal treatment selectively cleaved β-O-4 linkages since the ether linkage is the predominant linkages in lignin S units [30]. In needs to be pointed out that for accurate calculation of S/G ratio in wheat straw, the 4-ethylguaiacol (peak 10) and 4-vinylsyringol (peak 21) were excluded. Since the ferulic acids (FA) are not important in wood [21], the S/G ratio was not adjusted for these vinyl products in poplar.

Fungal degradation of the lignin side chain is also evidenced by Py-GC/MS. This indicates the decrease in the phenolic compounds bearing 3 C-atoms (ph-C3) and the concomitant increase of the phenolic compounds bearing 1 to 2 C-atoms (ph-C1-2) [31, 34]. As a result, the ph-C1-2/ph-C3 ratio increased in fungal-treated poplar and wheat straw and further increasing was observed in Mn addition samples (Fig. 2C). The increase in ph-C1-2/ph-C3 ratio was mainly determined by the decrease in ph-C3 products. The decrease of these products was more pronounced in Mn addition samples (Additional file 1: Table S1 and S2). Under pyrolysis conditions, lignin decomposed to aromatic products with various chain-lengths (ph-C0, ph-C1, ph-C2 and ph-C3) through bond cleavage [36, 37]. Clearly, the longest chain length of the products after pyrolysis equals the side chain lengths of the structures they originate from. Thus, ph-C3 can only form the subunits initially with three carbon side chains. In lignocellulosic biomass, the maximum inter-unit linkages (β-O-4) consist with C_α_-C_β_-C_γ_ side chains [38], thus, most of the ph-C3 products during pyrolysis were from the cleavage of β-O-4 linkages. As the result, the decreased content of ph-C3 products provides the indirect evidence of the reduced portion of β-O-4 linkages in biomass treated by fungi with Mn addition.

Moreover, in both treated poplar and wheat straw with Mn addition the C_α_-oxidized phenolic compounds such as aromatic ketones, aldehydes and acids were significantly increased during fungal treatment (Fig. 2D). In the case of poplar, the percentage of the C_α_-oxidized compounds was 7.16% in raw poplar, initially, and increased from 11.61, 8.93 and 8.56% in the fungal-treated samples up to 20.96, 9.23 and 9.61% in Mn-assisted fungal-treated poplar, separately. Similarly, in the case of wheat straw, the percentage of the C_α_-oxidized compounds was 5.44% in the control sample. It also slightly increased from 8.45, 6.17 and 7.38% in the fungal-treated wheat straw samples up to 8.46, 6.51 and 7.61%, respectively, with Mn-assisted treatment. The increased C_α_-oxidized compounds found in fungal-treated biomass have been considered as being indicative of oxidative alteration of lignin side chains at C_α_ position [31, 34, 35]. Moreover, the abundance of vanillic acid methyl ester (peak 21 in poplar and peak 22 in wheat straw) was found increased by Mn addition during fungal treatment (Additional file 1: Table S1 and S2). An increase in vanillic acid methyl ester was also the evident in the degraded biomass, suggesting C_α_–C_β_ oxidative cleavage of β-ether during fungal degradation [35]. The overall Py-GC/MS results indicated that Mn addition enhanced selective delignification and oxidative cleavage of β-O-4 linkages in fungal-treated biomass.

### Whole cell wall 2D HSQC NMR

**Fig. 3:**
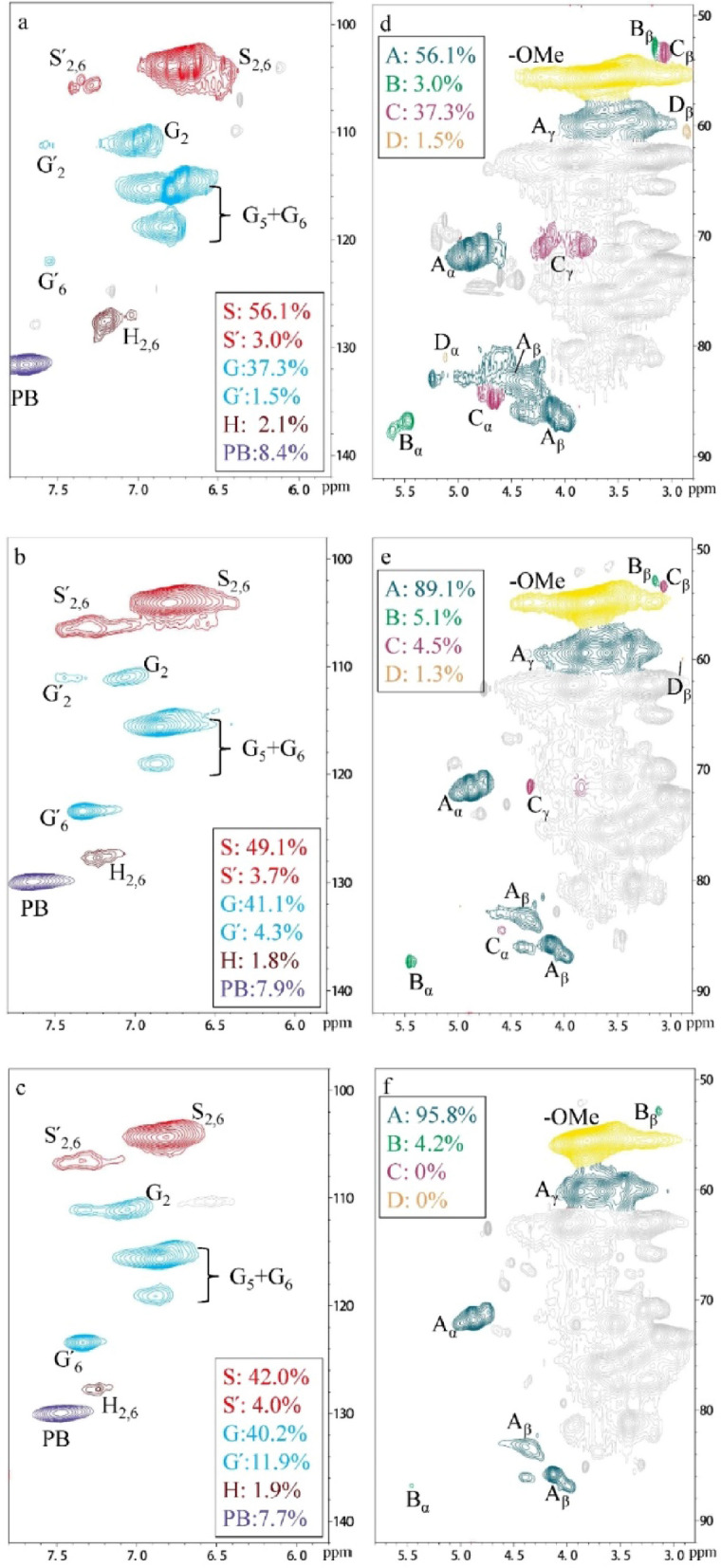
2D HSQC NMR spectra of raw, *Pc*-treated and *PcM*-treated poplar. a, b and c aromatic (δ_C_/δ_H_ 100–140/6.0–8.0) region, d, e and f aliphatic (δ_C_/δ_H_ 50–90/2.5–6.0) region. Unassigned, amino acid and carbonhydrate residues are shown in gray. See Additional files for spectra of other fungal-treated lignocelluosic feedstocks (Additional file 1: Figure S4 and S5). The annotated substructures of correlated peaks are shown in Additional file 1: Figure S6

To gain a detailed information about bond cleavage during Mn-assisted fungal treatments, 2D HSQC NMR spectroscopy was conducted. The main advantage of this approach is that it can be used to analyze whole cell wall of lignocellulose, thus, does not need to separate or depolymerize material except for the ball milling, which did not affect the inter-unit linkages of lignin [33, 39]. The NMR speatra of raw, *Pc*-treated and *PcM*-treated poplar included aromatic (δ_C_/δ_H_ 100–140/6.0–8.0) region and aliphatic (δ_C_/δ_H_ 50–90/2.5–6.0) region (Fig. 3). The chemical shift assignments of ^1^H–^13^C correlations for lignin are listed in Additional file 1: Table S3. The HSQC spectra of poplar treated by other fungus and wheat straw raw and treated by all three fungi are shown in Additional file 1: Figures S4 and S5, respectively. The correlated assigned substructres of the spectra are presented in Additional file 1: Figure S6. Table 2 summarizes the relative abundance of lignin subunits and bimass inter-unit linkages in the biomass samples.

**Table 2:** Structural characterization in the HSQC spectra of poplar and wheat straw

Treatment	Lignin subunits			Lignin inter-unit linkages^b^				Condensation degree^c^
	S/G	S′ + G′ (%)^a^	FA (%)^a^	β-O-4′	β-5′	β-β′	β-1′	
Poplar								
Raw	2.969	4.5	–	0.132	0.007	0.012	0.005	0.182
* Pc*	2.244	8.0	–	0.114	0.007	0.006	0.002	0.122
* Po*	2.502	4.9	–	0.117	0.003	0.010	0.002	0.129
* Pv*	2.487	5.4	–	0.121	0.014	0.005	0.001	0.161
* PcM*	1.689	15.9	–	0.098	0.004	ND	ND	0.044
* PoM*	2.244	5.0	–	0.102	0.000	0.008	0.001	0.085
* PvM*	2.103	5.9	–	0.109	0.008	0.004	0.001	0.115
Raw	1.421	4.1	12.3	0.207	0.016	0.008	0.004	0.138
* Pc*	1.426	6.1	9.6	0.182	0.006	0.005	ND	0.060
* Po*	1.202	3.6	1.4	0.152	0.004	0.005	ND	0.059
* Pv*	1.381	5.7	8.3	0.179	0.009	0.005	ND	0.081
* PcM*	1.430	9.5	9.4	0.176	0.005	0.004	ND	0.050
* PoM*	1.146	3.9	0.9	0.135	0.002	0.003	ND	0.036
* PvM*	1.367	8.6	7.6	0.156	0.004	0.004	ND	0.056

*ND* not detected

^a^Percentage of subunits vs (S + G + H)

^b^Linkages are relative to the methoxy

^c^Ratio of (β-5′ + β-β′ + β-1′)/ β-O-4′

In the aliphatic region of NMR spectra, distribution of inter-unit linkages in lignin structure was provided. Similar to studies by others [40, 41], β-O-4′ ether (A) is the most aboundent inter-unit linkages in poplar and wheat straw (Table 2). Meanwhile, the signals from β-5′ (phenylcoumarans, B), β-β′ (resinols, C) and β-1′ (spirodienones, D) can be detected in the NMR spectrum (Fig. 3). The analytic results showed that the relative abundances of carbon–oxygen (C–O) and carbon–carbon (C–C) linkages in fungal-treated samples decreased compared to raw poplar and wheat straw, indicating bond cleavage occurred during fungal attack. Moreover, Mn addition further decreased abundances of all inter-unit linkages in treated poplar and wheat straw. A further inspection of the bond cleavegeshows that the cleavage of the recalcitant C–C bonds (β-5′, β-β′ and β-1′) were faster than the cleavage of weaker C–O bond (β-O-4′) during fungal treatment, leading to the decreased condensation degree ((β-5′ + β-β′ + β-1′)/ β-O-4′) (Table 2). The decreased condesation degree caused by fungal degradation indicated the reduced recalcitrance in the treated biomass [22]. Mn addtion further decreased the condensation degree of poplar from 0.122, 0.129 and 0.161 down to 0.044, 0.085 and 0.115 during *Pc*, *Po* and *Pv* attack, respectively. Similar degradation pattern was observed in fungal-treated wheat straw, that condensation degree decreased from 0.60, 0.59 and 0.081 down to 0.050, 0.036 and 0.056, separately. In particlular, the β-1′ and β-β′, the major C–C in wheat straw, disappeared after *PcM* treatment (Additional file 1: Figure S5). Compare to fungal solo-treated samples, more than 30% of the condensation degree further decreased in Mn-assisted fungal-treated biomass. The overall results indicated that Mn-assisted fungi breakdown more C–C bonds and makes the inter-unit network more incompact in the residual lignin, which resulted in less recalcitrance to enzymatic hydrolysis of Mn-assisted fungal-treated poplar and wheat straw.

Interestingly, the percentage of C_α_-oxidized subunits (S′ + G′) was clearly increased by Mn addition. Particularly, C_α_-oxidized subunits in treated wheat straw were more prone to accumulate than in treated poplar (Table 2). The abundance of C_α_-oxidized subunits increased by around 50% in wheat straw and about 10% in poplar after Mn added to fungal treatment. During the fungal treatment of lignocellulose, C_α_-oxidized products originated from oxidative cleavage of inter-unit linkages in lignin [33]. The C_α_-oxidized products accumulated in the residual lignin could be also evidenced by the reduced amount of intact inter-unit linkages in treated lignin. Thus, Mn-assisted fungus degraded more lignin inter-unit linkages through oxidative cleavage.

In the aromatic region, typical signals in poplar lignin were observed (S_2,6_, G_2_, G_5_, G_6_, H_2,6_, and PB, Fig. 3), while *p*-coumaric acids (*p*CA) and FA-related signals usually shown in wheat straw lignin were also observed (Additional file 1: Figure S5). Similar to the Py-GC/MS analysis, a decrease in the S/G ratio from 2.969 to 2.244, 2.502, 2.487, and further down to 1.689, 2.244, 2.103 was observed in raw, *Pc*, *Po*, *Pv*-treated, and *PcM*, *PoM*, *PvM*-treated poplar (Table 2), respectively. Meanwhile, a decrease in the S/G ratio was observed in treated wheat straw, expect a slight increase in *Pc* and *PcM*-treated wheat straw. These NMR results also indicated the preferential removal of S units in fungal-treated samples. Both *p*CA and FA were excluded when calculating the lignin subunits of wheat straw in NMR. Another clear result from NMR was the decrease of FA in wheat straw after fungal treatment, especially FA abundance further decreased in Mn-assisted fungal-treated wheat straw. The percentage of FA decreased from 12.3% to 9.6, 1.4 and 8.3% in the *Pc*, *Po*, *Pv*-treated wheat straw, and further decreased down to 9.4, 0.9 and 7.6% in the *PcM*, *PoM*, *PvM*-treated wheat straw, separately. In the plant cell wall of wheat straw, FA linked lignin and arabinoxylans/xylose via ester-linkages, forming a typical cross-linked LCC structure [21]. This structure was shown to play a very important role in affecting the digestibility of lignocellulose [42, 43]. Such structure in biomass could be cleaved by extracellular enzymes secreted by fungus [8, 33], resulting the cleavage of LCCs linkage bridged by FA during fungal degradation of lignocellulose [8, 22]. Fungal cleavage of the FA-ester linkages resulted in the reduction of relative abundances of FA in the LCCs [22]. In this study, the enhanced degradation of FA was in accord with the selective lignin and xylose reduction during Mn-assisted fungal treatment (Table 1). In contrast to wheat straw LCCs, the FA motif is not an important subunit in poplar LCCs with a relative minor abundance, which cannot be detected in poplar (Fig. 3). It is difficult to identify LCCs in biomass especially in woody biomass, due to the heavy overlapping of signals related to carbohydrates [44]. For more evidence contact with LCCs biodegradation, future study with LCCs isolation from biomass may needed.

### Correlations between structural features and biomass digestibility

**Fig. 4:**
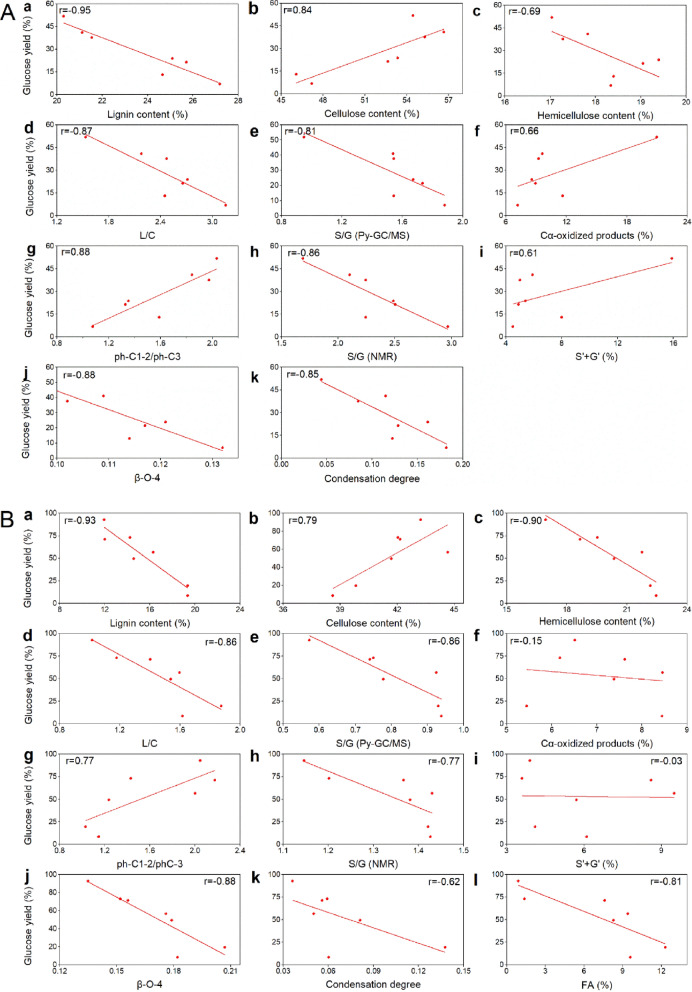
Correlation between glucose yield and biomass-related factors. Lignin content a, hemicellulose content b, cellulose content c, L/C ratio d, S/G ratio (Py-GC/MS) e, C_α_-oxidized products f, ph-C1-2/ph-C3 ratio g, S/G ratio (NMR) h, S′ + G′ i, β-O-4 linkages j, condensation degree k, and FA l (only in wheat straw): poplar A and wheat straw B

It is well-known that the removal of recalcitrance in lignocellulose during fungal degradation is mainly via bond cleavage [8, 33]. Thus, the linear relationships between physicochemical and structural features and glucose yield were investigated (Fig. 4). The biomass features including lignin, cellulose, and hemicellulose content, L/C ratio, S/G ratio (Py-GC/MS), C_α_-oxidized products, ph-C1-2/ph-C3 ratio, S/G ratio (NMR), S′ + G′, FA, β-O-4 linkages and condensation degree were used for analysis. The results showed that the changes in physicochemical and structural features in poplar and wheat straw have the similar impact on glucose yields during fungal treatments. The results showed that lignin content was strongly negatively (− 0.95 < *r* < − 0.90) correlated with glucose yields in poplar and wheat straw Meanwhile, the hemicellulose content was moderately negatively (*r* = − 0.69) correlated with glucose yields in poplar and strongly negatively (*r* = − 0.90) in wheat straw. The cellulose content was moderately positively correlated (0.75 < *r* < − 0.85) with glucose yields in poplar and wheat straw. The L/C ratio, S/G ratio and β-O-4 linkages were strongly negatively (− 0.90 < *r* < − 0.80) correlated with glucose yield in poplar and wheat straw. The results indicated that enhanced selective delignification caused by preferential lignin S unit degradation and β-O-4 cleavage during fungal treatment led to reduced recalcitrance to enzyme access in the residue biomass. It in turn resulted in the increase in biomass digestibility. Moreover, FA was strongly negatively (*r* = − 0.81) correlated with glucose yield in wheat straw. This is consistent with other reports showing that FA-related LCCs play a crucial role in affecting the digestibility of wheat straw [8]. The cleavage of lignin–ether–ferulic acid–ester–hemicellulose linkage in grass biomass lignin by white rot fungus was also reported recently [22]. The results also explain the reason hemicellulose content in wheat straw poplar (Fig. 4B, c) was more related to glucose yield than in poplar (Fig. 4A, c). The condensation degree was strongly negatively (*r* = − 0.88) correlated with glucose yield in poplar, but moderately negatively (*r* = − 0.65) correlated with glucose yield in wheat straw. This result showed that besides ether cleavage, the effect of C–C linkages degradation on cellulose accessibility is important. It is interesting to observe that the accumulation of C_α_-oxidized products and S′ + G′ were moderately positive (*r* = 0.66 and 0.61) in glucose yield of the poplar samples. However, there were no correlations between C_α_-oxidized products and S′ + G′ with glucose yield in wheat straw. The fact that these oxidized produces could be further metabolized by fungus makes these products not a good indicator to lignin degradation during fungal attack [30], and thus led to a random correlation between oxidized produces and glucose yield.

### PCA for correlations to biomass digestibility

**Fig. 5:**
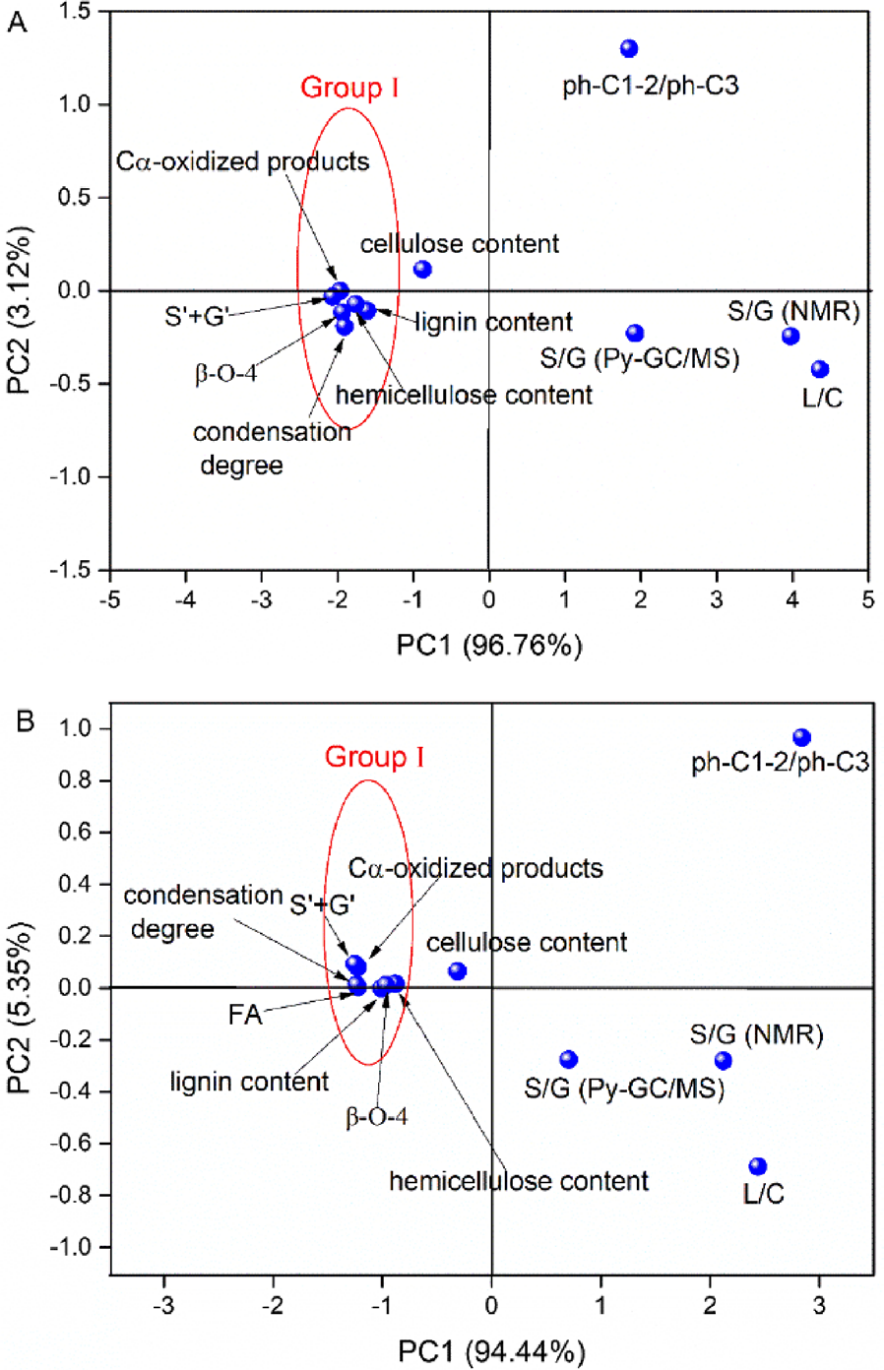
Correlation plots of PCA for the chemical and structural factors of lignocellulose. Poplar A and wheat straw B

In this study, various chemical and structural features have been proven to play important roles in affecting the biomass digestibility of fungal-treated poplar and wheat straw. PCA reduces the dimensionality of the factors from large multivariate data, thus elucidating the nonlinear and comprehensive factors affecting the digestibility of biomass [26, 27]. The similarity and difference between these factors simply determined by the loading values in plots [27]. The factors related to glucose yield and the plots with their loading values are shown in Fig. 5. These factors were lignin content, cellulose content, hemicellulose content, L/C ratio, S/G ratio (Py-GC/MS), C_α_-oxidized products, ph-C1-2/ph-C3 ratio, S/G ratio (NMR), S′ + G′, FA, β-O-4 linkages, and condensation degree. The two principal components of glucose in poplar and wheat straw explained 99.88% and 99.79% of the total variance, respectively, and be used to construct two-dimensional plots for visualization. In particular, the PC1 (96.76% and 94.44%) that explained most of the total variance and the loading value on PC1 could be sufficient to describe the similarities and differences of the factors.

As shown in Fig. 5, the similarities and differences between the physicochemical and structural properties were characterized well by PC1. The related loading values are shown in Table 3. The factors lignin content, hemicellulose content, C_α_-oxidized products, S′ + G′, FA (wheat straw), β-O-4 linkages, and condensation degree could be classified as Group I, however, other factors could not be classified in the groups. Meanwhile, the loading values of factors in the group were similar, indicating their same level of contribution to PC1. Interestingly, the factors in Group I all had negative effects on enzymatic hydrolysis. The result indicated that these factors had equal contribution to the biomass polysaccharides conversion. The results indicated that Mn addition to fungal treatment through the deconstruction of the lignocellulosic matrix via selectively lignin and hemicellulose removal, which is mainly caused by the enhanced cleavages the β-O-4 linkages in poplar and wheat straw lignin via C_α_-oxidative reactions, as a result, the condensation degree in lignin was significantly reduced. Meanwhile, the enhanced decreasing of FA, β-O-4 and hemicellulos content while increased glucose yield was observed in Mn-assisted treated wheat straw, and those factors were proven strongly negative to glucose yield, indicating that deconstruction of wheat straw LCCs by fungus via lignin–ether–ferulic acid–ester–hemicellulose linkage was important in reducing its recalcitrance. Consequently, the fungal treatment greatly reduces the recalcitrance of biomass and increases the enzyme accessibility of the remaining cellulose, leading to increases in glucose yield.

**Table 3:** The component coefficients of glucose yield-relevant factors according to the PCA

Glucose yield	Poplar		Wheat straw	
	Component		Component	
	1	2	1	2
S′ + G′	− 2.06	− 0.03	− 1.25	0.09
C_α_-oxidized products	− 1.97	0.00	− 1.22	0.08
β-O-4	− 1.94	− 0.12	− 0.97	0.01
Condensation degree	− 1.91	− 0.19	− 1.24	0.01
FA	−	−	− 1.22	0.00
Hemicellulose content	− 1.76	− 0.07	− 0.88	0.02
Lignin content	− 1.61	− 0.11	− 1.01	0.00
Cellulose content	− 0.87	0.11	− 0.31	0.06
S/G ratio (Py-GC/MS)	1.93	− 0.23	0.71	− 0.28
Ph-C1-2/ph-C3	1.85	1.30	2.84	0.97
S/G ratio (NMR)	3.97	− 0.24	2.12	− 0.28
L/C	4.36	− 0.42	2.44	− 0.69

## Conclusions

The broad-spectrum of Mn addition on enhancing the enzymatic hydrolysis of woody and grass lignocellulose was investigated. Over 90% of glucose yield was successfully achieved in wheat straw after Mn-assisted *P. ostreatus* treatment. The role of Mn addition in improving fungal treatment efficiency was evaluated by comparing the cross-linked structure of biomass with and without Mn addition. The structural features of treated biomass as determined by Py-GC/MS and 2D HSQC NMR confirmed that, Mn-assisted fungal-treated lignin was high in C_α_-oxidized products and the β-O-4 ether linkages was lower than fungal solo-treated lignin. Furthermore, the correlation analysis PCA demonstrated that Mn addition to fungal treatment enhanced biomass sugar conversion via C_α_-oxidation cleavage of β-O-4 ether linkages and further led to reduced condensation degree in lignin, as a result, the biomass recalcitrance was significantly decreased. Moreover, the deconstruction of LCCs was also important in reducing wheat straw recalcitrance. The relationship between structure and biomass conversion provides a new insight in reducing recalcitrance of the biomass during fungal treatment for the purpose of developing environmentally friendly treatment for biorefinery using lignocellulose.

## Materials and methods

### Feedstocks and fungus preparation

Wheat straw and poplar wood were obtained from Grange Supply Co. (Pullman, WA, USA). The biomass was chopped into pieces and milled to 0.5–0.3 mm particles, then dried in an oven at 50 °C for 3 days to remove 95% of water. Dried biomass was then cooled in a desiccator at room temperature for further use.

White rot fungus *Phanerochaete chrysosporium* was obtained from Ohio State University (Wooster, OH, USA). *Pleurotus ostreatus* was obtained from Culture Collection Center of Huazhong Agricultural University (Wuhan, Hubei, China). *Physisporinus vitreus* (GenBank No. KU958584) was isolated from a stone seam at the Huazhong University of Science and Technology (Wuhan, Hubei, China). The strains were cultured on potato dextrose agar plat plates for 7 days at 28 °C. Ten discs (1 cm in diameter) of the plate culture were filled in a 500-ml Erlenmeyer flask containing 50 ml of potato dextrose medium and cultivated under stationary condition for 7 days. The grown mycelium was filtered, washed with sterile water, and then blended with 50 ml of sterile water to obtain a homogeneous mycelium for biomass inoculation.

### Fungal pretreatment

Fungal pretreatment was performed in 250-ml flasks with 3 g of dried biomass and liquid medium (deionized (DI) water or 0.676 g/L MnSO_4_·H_2_O) to obtain 71% moisture content. The prepared biomass was then sterilized at 121 °C for 20 min, cooled to room temperature, and then inoculated with 2 ml of homogeneous mycelium of fungi. Fungal treatments were performed at 28 °C for the fermentation time of 28 days. The 28-day-treated biomass was dried at 50 °C for 3 days and then used for subsequent analysis. All the fungal treatments have three replicates. The moisture content, amount of Mn addition (0.01 mM/g biomass) and treatment time were chosen based on the optimal results of previous experiments [9, 15].

### Enzymatic hydrolysis

Enzymatic hydrolysis of biomass was conducted in a 250-ml Erlenmeyer flask. The commercial enzymes Cellic^®^ CTec 2 and HTec 2 (Novozymes, Bagsvaerd, Denmark) were used at a protein concentration of 20 mg and 2 mg of protein/g of cellulose, at a solid loading of 2% in citrate buffer (50 mM, pH 4.8). The cellulase activity was 30 filter paper unit (FPU)/g cellulose for CTec 2 and was determined by previously assay method [45]. HTec 2 was dosed 10% of CTec 2 in this study. To inhibit the growth of bacteria during hydrolysis process, sodium azide was added to the citrate buffer to make a final concentration to 0.5 ‰ (w/v). The enzymatic hydrolysis was performed at 50 °C for 48 h. The mono-sugars in supernatant were collected and analyzed by ion exchange chromatography (IC, Dionex ICS-3000 IC, MA, USA) equipped with an electrochemical detector and a CarboPac PA20 Guard (3 × 30 mm) column (Dionex, MA, USA). The temperature of the column was maintained at 30 °C and the mobile phase flow rate was 0.5 ml/min. The samples were carried by 80% double-deionized water and 20% 52 mM NaOH. The sugar contents were quantified against the calibration curve by running standard solutions ranging from 1 to 20 ppm for each sugar species of interest. Glucose and xylose yields were calculated according to the following equations:

1 $$\mathrm{Glulose\,yield }\left(\mathrm{\%}\right)=\frac{\mathrm{Glucose\,amount }(\mathrm{mg})}{\mathrm{Cellulose\,amount }(\mathrm{mg})\times 1.11}\times 100,$$

2 $${\rm{Xylose}}{\mkern 1mu} {\rm{yield}}\left( \% \right) = \frac{{{\rm{Xylose}}.{\rm{amount}}({\rm{mg}})}}{{{\rm{Hemicellulose}}{\mkern 1mu} {\rm{amount}}({\rm{mg}}) \times 1.14}} \times 100,$$

where 1.11 and 1.14 are conversion factors for polysaccharide to monosaccharide [46].

### Composition analysis

The chemical composition of the biomass was determined based on the protocol from the National Renewable Energy Laboratory [46]. Biomass samples were hydrolyzed with 72% (w/w) sulfuric acid at 30 °C for 1 h, then immediately diluted to 4% (w/w) using DI water. The diluted samples were acid hydrolyzed with 4% acid at 121 °C for 1 h. Acid-soluble lignin in the liquid phase was determined by UV–vis under 320 nm (wheat straw) and 240 nm (poplar), respectively. Mono-sugars in aqueous phase were analyzed by IC. Acid-insoluble lignin and ash as solid residues were measured by weight using Mettler Toledo AG204 balance (Columbus, OH, USA).

### Py-GC/MS analysis

Py-GC/MS is a rapid and semi-quantitative method for characterizing the chemical structure of lignin in biomass. Pyrolysis was carried out using a CDS Pyroprobe 5000 autosampler (Oxford, PA, USA) coupled to a Thermo Trace GC 6890N/MSD 5975B gas chromatography/mass spectrometry system (Bellevue, WA, USA). Samples were weighed using a Mettler Toledo AX205 DeltaRange analytic balance (Columbus, OH, USA). Approximately 1 mg of sample was loaded into a quartz tube and gently packed with quartz wool prior to pyrolysis. Pyrolysis of samples was performed at 500 °C for 1 min with the interface temperature of 250 °C. The pyrolyzed vapors were separated by means of a 30 m × 0.25 μm inner diameter (5%-phenyl)-methylpolysiloxane non-polar column with helium gas flow rate at 1 ml/min and via a split ratio of 50:1. The GC was programmed at a linear heating (6 °C /min) from 40 to 280 °C, and the oven was held at 280 °C for 10 min. The mass spectrometer was operated in EI mode (70 eV) at a source temperature of 230 °C. Pyrolyzed lignin compounds were identified by comparing retention time and mass spectrum with standards and published data [31–33]. The spectra of raw biomass (as the example) with numbered peaks (lignin pyrolytic products) are shown in Additional file 1: Figure S2. The structures of phenolic pyrolysis compounds are shown in Additional file 1: Figure S3. The identities and relative abundances of lignin compounds from raw, fungal-treated and fungal–Mn-treated biomass were assigned according to the previous reports (Additional file 1: Table S1 and S2) [31, 33–35, 47].

### Whole cell wall 2D HSQC NMR spectroscopy

The raw biomass (wheat straw and poplar), fungal-treated biomass and biomass treated by fungal with Mn addition (fungal-Mn) was grounded (0.1 mm) and Soxhlet extracted with ethanol–benzene mixed solvent (1/2, v/v) at least 48 h until the solvent became clear, and then dried the samples in the chemical hood. 100 °C HPLC-grade water was used to treat the samples, and the water was changed every hour until it became clear. The extractive-free residues were freeze-dried and then milled in a Retsch PM 100 planetary ball mill (Retsch, Haan, Germany). About 0.5 g of sample was added along with zirconium dioxide (ZrO_2_) to a 50-ml vessel and milled with ten 10-mm ZrO_2_ balls at 500 rpm with the total milling time of 10 h. Every 15 min of mill and 10 min of pause were alternative operated. Approximately 100 mg of ball-milled sample was suspended in 800 µL of DMSO-*d*_*6*_ in the1.5 ml NMR tube.

The HSQC spectra were recorded at 25 °C and on an Agilent DD2 600 MHz superconducting NMR spectrometer with “gHSQCAD” pulse sequence, the experimental procedure of HSQC based on previously published paper [48]. ^1^H and ^13^C spectral widths were 5000 Hz and 25,000 Hz, respectively. The number of collected complex points was 2048 for the ^1^H dimension, with a recycle delay of 1.75 s. The number of collected scans was 64 and 256 times increment were recorded in the ^13^C dimension. The ^1^J_CH_ used was 140 Hz. The central DMSO peak (δ_C_/δ_H_ 9.5/2.49 ppm) was used as reference for chemical shifts. Correlation peaks of HSQC were assigned according to previous literatures (Additional file 1: Table S5) [38, 39, 49, 50]. Semiquantitative analysis of the integrals of the HSQC correlation peaks was performed using MestreNova software (version 6.0.1) [38, 49]. In the aliphatic oxygenated region, the inter-unit linkages of lignin were calculated based on the methoxyl contour [40, 49]. Integrals of phenylcoumaran and resinol were estimated from the C_α_-H_α_ correlations. In the aromatic region, the relative abundances of S, G, H units and the *p*-hydroxy-benzoate (PB), FA and *p*CA were estimated from their respective C_2_–H_2_ correlations.

### Statistical analysis

The factors including L/C ratio, S/G ratio (Py-GC/MS), C_α_-oxidized products, ph-C1-2/ph-C3 ratio, S/G ratio (NMR), S′ + G′, FA, β-O-4 linkages and condensation degree were analyzed by correlation analysis and principal component analysis using R 3.5.1 software [51]. The principal components are linear combinations of the original variables, and in particular, the eigenvectors of the covariance matrix of the variables. In principal component regression, the components that account for the majority of variance in the data are used as variables [52].

## Supplementary Information

**Additional file 1**:** Table S1**. Relative abundance of the phenolic compounds derived peaks identified in the Py-GC/MS of poplar.** Table S2**. Relative abundance of the phenolic compounds derived peaks identified in the Py-GC-MS of wheat straw.4 Table S3. The assignments of 13C-1H peaks in HSQC spectra.** Figure S1**. Weight loss of poplar and wheat straw for different treatments.** Figure S2**. Py-GC/MS profiles of raw poplar A and wheat straw B.**Figure S3**. Structures of the wheat straw (poplar) lignin derived compounds released from the Py-GC/MS.** Figure S4**. 2D HSQC NMR spectra of fungal-treated poplar.** Figure S5**. 2D HSQC NMR spectra of raw and fungal-treated wheat straw. ** Figure S6**. HSQC NMR annotated structure.

## Data Availability

The datasets used and/or analyzed during the current study are available from the corresponding author on reasonable request.
