# Supplementary material for: A comprehensive study of the promoting effect of manganese on white rot fungal treatment for enzymatic hydrolysis of woody and grass lignocellulose
Source: Biotechnol Biofuels. 2021 Sep 6;14:176. doi: 10.1186/s13068-021-02024-7 (PMC8420007; doi:10.1186/s13068-021-02024-7)

Additional file 1

A comprehensive study of the promoting effect of manganese on white rot fungal treatment for enzymatic hydrolysis of woody and grass lignocellulose

Xiao Fu^a1^, Jialong Zhang^a1^, Xiangyu Gu^b^, Hongbo Yu^a*^, Shulin Chen^b*^

^a^Key Laboratory of Molecular Biophysics of MOE, College of Life Science and Technology, Huazhong University of Science and Technology, Wuhan, 430074, China

^b^Department of Biological Systems Engineering, Washington State University, Pullman, WA 99164, USA

Contact information:

yuhongbo@hust.edu.cn

[Table S1. Relative abundance of the phenolic compounds derived peaks identified in the Py-GC/MS of poplar. 2](#_Toc67307194)

[Table S2. Relative abundance of the phenolic compounds derived peaks identified in the Py-GC-MS of wheat straw. 4](#_Toc67307195)

[Table S3. The assignments of 13C-1H peaks in HSQC spectra. 6](#_Toc67307196)

[Figure S1. Weight loss of poplar and wheat straw for different treatments. 7](#_Toc67307197)

[Figure S2. Py-GC/MS profiles of raw poplar A and wheat straw B. 8](#_Toc67307198)

[Figure S3. Structures of the wheat straw (poplar) lignin derived compounds released from the Py-GC/MS. 9](#_Toc67307199)

[Figure S4. 2D HSQC NMR spectra of fungal-treated poplar. 10](#_Toc67307200)

[Figure S5. 2D HSQC NMR spectra of raw and fungal-treated wheat straw. 12](#_Toc67307201)

[Figure S6. HSQC NMR annotated structure. 15](#_Toc67307202)

# Table S1. Relative abundance of the phenolic compounds derived peaks identified in the Py-GC/MS of poplar.

| No. | Compound | Structure feature^a^ | Side chain length^b^ | Raw | *Pc* | *Po* | *Pv*^d^ | *PcM* | *PoM* | *PvM* |
| --- | --- | --- | --- | --- | --- | --- | --- | --- | --- | --- |
|  |  |  |  |  |  |  |  |  |  |  |
| 1 | Phenol | H | 0 | 9.99 ± 0.77 | 8.36 ± 0.30 | 9.87 ± 0.42 | 10.83 ± 1.15 | 7.10 ± 1.96 | 10.58 ± 0.57 | 11.23 ± 1.63 |
| 2 | Phenol, 2-methyl | H | C_α_ | 1.37 ± 0.03 | 1.36 ± 0.35 | 1.74 ± 0.09 | 1.18 ± 0.55 | 1.78 ± 0.39 | 1.59 ± 0.19 | 1.55 ± 0.14 |
| 3 | Phenol, 3-methyl | H | C_α_ | 1.02 ± 0.22 | 1.11 ± 0.32 | 1.36 ± 0.05 | 1.25 ± 0.31 | 1.67 ± 0.30 | 1.11 ± 0.12 | 1.05 ± 0.26 |
| 4 | Guaiacol | G | 0 | 5.22 ± 0.08 | 5.37 ± 0.32 | 6.19 ± 0.18 | 5.93 ± 0.04 | 8.61 ± 0.60 | 6.59 ± 0.03 | 7.35 ± 0.57 |
| 5 | 4-ethylphenol | H | C_β_ | 0.39 ± 0.03 | 0.39 ± 0.04 | 0.43 ± 0.04 | 0.35 ± 0.08 | 0.44 ± 0.13 | 0.33 ± 0.03 | 0.29 ± 0.09 |
| 6 | 2,3-dihydroxybenzaldehyde | Mis.^c^ | 0 | 0.79 ± 0.15 | 1.52 ± 0.83 | 0.95 ± 0.03 | 1.46 ± 0.82 | 1.27 ± 0.04 | 1.19 ± 0.59 | 1.04 ± 0.29 |
| 7 | 4-methylguaiacol | G | C_α_ | 2.98 ± 0.10 | 3.81 ± 0.48 | 3.79 ± 0.32 | 3.28 ± 0.04 | 3.08 ± 0.30 | 3.79 ± 0.17 | 3.52 ± 0.70 |
| 8 | 3-methoxycatechol | Mis. | 0 | 2.12 ± 0.62 | 2.65 ± 0.73 | 1.95 ± 0.39 | 2.98 ± 1.16 | 2.84 ± 0.22 | 2.38 ± 0.51 | 4.06 ± 0.87 |
| 9 | 4-ethylguaiacol | G | C_β_ | 1.65 ± 0.21 | 1.96 ± 0.25 | 1.61 ± 0.14 | 2.20 ± 0.54 | 1.80 ± 0.14 | 2.06 ± 0.12 | 2.21 ± 0.32 |
| 10 | 4-vinylguaiacol | G | C_β_ | 6.80 ± 0.50 | 6.64 ± 0.45 | 5.81 ± 0.10 | 5.74 ± 0.23 | 5.88 ± 0.24 | 6.03 ± 0.32 | 4.99 ± 0.55 |
| 11 | Syringol | S | 0 | 9.54 ± 1.14 | 7.70 ± 0.52 | 9.96 ± 0.18 | 9.64 ± 0.43 | 8.73 ± 0.80 | 10.41 ± 0.9 | 9.96 ± 0.99 |
| 12 | Eugenol | G | C_γ_ | 1.89 ± 0.08 | 1.36 ± 0.14 | 1.57 ± 0.13 | 1.59 ± 0.24 | 1.54 ± 0.34 | 1.96 ± 0.93 | 1.18 ± 0.15 |
| 13 | Vanillin | G | C_α_ | 0.86 ± 0.28 | 1.58 ± 0.25 | 1.27 ± 0.29 | 1.14 ± 0.03 | 2.59 ± 0.10 | 1.13 ± 0.17 | 1.29 ± 0.29 |
| 14 | Cis-isoeugenol | G | C_γ_ | 1.52 ± 0.14 | 1.55 ± 0.13 | 1.25 ± 0.24 | 1.18 ± 0.01 | 1.28 ± 0.22 | 1.30 ± 0.18 | 1.23 ± 0.11 |
| 15 | 4-methylsyringol | S | C_α_ | 5.22 ± 1.86 | 5.89 ± 1.19 | 6.01 ± 0.33 | 5.70 ± 0.85 | 3.13 ± 0.39 | 5.80 ± 0.14 | 4.61 ± 0.30 |
| 16 | Trans-isoeugenol | G | C_γ_ | 3.66 ± 1.20 | 2.25 ± 0.49 | 2.86 ± 0.37 | 3.00 ± 1.22 | 2.04 ± 0.19 | 3.28 ± 0.34 | 2.54 ± 0.24 |
| 17 | Homovanillin | G | C_β_ | 1.16 ± 0.11 | 1.46 ± 0.08 | 1.18 ± 0.07 | 1.05 ± 0.23 | 1.47 ± 0.20 | 0.93 ± 0.09 | 0.86 ± 0.12 |
| 18 | 4-ethylsyringol | S | C_β_ | 2.99 ± 0.36 | 2.01 ± 0.27 | 2.88 ± 0.27 | 3.22 ± 1.57 | 1.39 ± 0.08 | 3.73 ± 0.47 | 3.31 ± 0.87 |
| 19 | Guaiacylacetone | G | C_γ_ | 2.58 ± 0.71 | 2.30 ± 0.36 | 2.26 ± 0.15 | 2.50 ± 0.92 | 2.82 ± 0.34 | 2.57 ± 0.35 | 3.18 ± 0.65 |
| 20 | 4-vinylsyringol | S | C_β_ | 10.07 ± 0.67 | 13.00 ± 1.00 | 10.32 ± 0.99 | 10.59 ± 4.46 | 8.21 ± 0.95 | 8.47 ± 0.59 | 10.89 ± 4.61 |
| 21 | Vanillic acid methyl ester | G | C_α_ | 0 ± 0 | 3.22 ± 0.62 | 1.34 ± 0.39 | 1.33 ± 0.23 | 8.69 ± 0.50 | 2.74 ± 0.70 | 2.40 ± 0.94 |
| 22 | Vanilloyl acetaldehyde | G | C_γ_ | 2.70 ± 0.09 | 2.24 ± 0.08 | 2.46 ± 0.09 | 2.27 ± 0.14 | 2.50 ± 0.21 | 2.19 ± 0.08 | 1.80 ± 0.28 |
| 23 | 4-propylsyringol | S | C_γ_ | 0.47 ± 0.09 | 0.51 ± 0.10 | 0.49 ± 0.11 | 0.52 ± 0.12 | 0.33 ± 0.29 | 0.55 ± 0.05 | 0.52 ± 0.08 |
| 24 | Cis-4-propenylsyringol | S | C_γ_ | 2.22 ± 0.57 | 1.85 ± 0.02 | 2.36 ± 0.21 | 1.63 ± 0.24 | 1.39 ± 0.20 | 2.36 ± 0.12 | 2.09 ± 0.50 |
| 25 | Syringaldehyde | S | C_α_ | 3.01 ± 0.14 | 3.24 ± 0.14 | 2.80 ± 0.12 | 2.54 ± 0.07 | 2.84 ± 0.27 | 2.60 ± 0.14 | 2.55 ± 0.11 |
| 26 | 4-propynesyringol | S | C_γ_ | 1.56 ± 0.08 | 0.72 ± 0.03 | 0.59 ± 0.09 | 0.35 ± 0.08 | 0.60 ± 0.31 | 0.44 ± 0.11 | 0.36 ± 0.15 |
| 27 | Trans-4-propenylsyringol | S | C_γ_ | 7.82 ± 0.45 | 6.14 ± 0.53 | 7.40 ± 0.22 | 7.13 ± 0.36 | 3.33 ± 0.06 | 6.88 ± 0.47 | 5.55 ± 0.74 |
| 28 | Homosyringaldehyde | S | C_γ_ | 3.14 ± 0.83 | 2.56 ± 0.04 | 2.10 ± 0.06 | 2.16 ± 0.04 | 2.25 ± 0.10 | 1.93 ± 0.22 | 2.12 ± 0.20 |
| 29 | Syringylacetone | S | C_γ_ | 2.07 ± 0.19 | 2.45 ± 0.41 | 2.34 ± 0.08 | 2.64 ± 0.43 | 3.01 ± 0.35 | 2.75 ± 0.13 | 3.27 ± 0.34 |
| 30 | Trans-coniferyl alcohol | G | C_γ_ | 0.59 ± 0.10 | 0.63 ± 0.12 | 0.61 ± 0.08 | 0.60 ± 0.16 | 0.31 ± 0.04 | 0.41 ± 0.20 | 0.38 ± 0.09 |
| 31 | Propiosyringone | S | C_γ_ | 0.64 ± 0.08 | 1.41 ± 0.02 | 1.13 ± 0.03 | 1.36 ± 0.06 | 2.52 ± 0.15 | 1.17 ± 0.07 | 1.66 ± 0.06 |
| 32 | Trans-sinapaldehyde | S | C_γ_ | 3.59 ± 0.42 | 2.76 ± 0.06 | 2.83 ± 0.22 | 2.66 ± 0.27 | 1.68 ± 0.13 | 2.26 ± 0.42 | 1.89 ± 0.22 |
| 33 | Trans-sinapyl alcohol | S | C_γ_ | 0.85 ± 0.27 | 0 ± 0 | 0.28 ± 0.04 | 0 ± 0 | 0 ± 0 | 0.17 ± 0.02 | 0 ± 0 |

Values are expressed as the means determined for three replicates ± SE.

The amount of each compound is expressed as percentages.

The compositional data were calculated based on H+G+S+mis.=100%.

^a^H:lignin H units, G:lignin G units, S: lignin S units.

^b^Lignin-derived phenols with 0, 1, 2 and 3 carbons in the side chain.

^c^Miscellaneous.

^d^Two replicates.

# Table S2. Relative abundance of the phenolic compounds derived peaks identified in the Py-GC-MS of wheat straw.

| No. | Compound | Structure feature^a^ | Side chain length^b^ | Raw | *Pc* | *Po* | *Pv* | *PcM* | *PoM* | *PvM* |
| --- | --- | --- | --- | --- | --- | --- | --- | --- | --- | --- |
|  |  |  |  |  |  |  |  |  |  |  |
| 1 | Phenol | H | 0 | 2.35 ± 0.87 | 2.86 ± 0.20 | 4.47 ± 0.25 | 3.86 ± 1.10 | 2.08 ± 0.05 | 4.62 ± 0.52 | 4.57 ± 0.25 |
| 2 | Phenol, 2-methyl | H | C_α_ | 2.51 ± 0.25 | 1.79 ± 0.22 | 3.29 ± 0.64 | 2.02 ± 0.96 | 1.37 ± 0.09 | 2.80 ± 0.42 | 2.54 ± 0.32 |
| 3 | Phenol, 3-methyl | H | C_α_ | 2.48 ± 0.40 | 2.25 ± 0.46 | 3.08 ± 0.79 | 3.13 ± 0.08 | 2.40 ± 0.19 | 3.11 ± 0.73 | 2.78 ± 0.41 |
| 4 | Guaiacol | G | 0 | 8.95 ± 0.18 | 8.62 ± 0.60 | 16.28 ± 0.64 | 15.62 ± 1.90 | 9.21 ± 0.34 | 20.64 ± 1.58 | 14.35 ± 0.58 |
| 5 | 4-ethylphenol | H | C_β_ | 1.11 ± 0.17 | 0.94 ± 0.05 | 1.78 ± 0.22 | 1.19 ± 0.59 | 0.62 ± 0.05 | 1.62 ± 0.14 | 1.18 ± 0.15 |
| 6 | 2,3-dihydroxybenzaldehyde | Mis.^c^ | 0 | 1.11 ± 0.03 | 1.23 ± 0.05 | 1.60 ± 0.24 | 1.36 ± 0.13 | 1.32 ± 0.07 | 1.71 ± 0.04 | 1.32 ± 0.19 |
| 7 | 4-methylguaiacol | G | C_α_ | 2.85 ± 0.40 | 2.86 ± 0.16 | 4.25 ± 0.57 | 2.80 ± 0.27 | 2.85 ± 0.47 | 3.48 ± 0.47 | 2.66 ± 0.28 |
| 8 | 4-vinylphenol | H | C_β_ | 10.53 ± 1.00 | 10.5 ± 0.97 | 4.19 ± 1.28 | 4.86 ± 1.93 | 7.87 ± 0.24 | 2.86 ± 0.51 | 4.67 ± 0.69 |
| 9 | 3-methoxycatechol | Mis. | 0 | 1.15 ± 0.06 | 1.73 ± 0.12 | 2.71 ± 0.45 | 3.07 ± 0.92 | 2.29 ± 0.50 | 2.61 ± 0.46 | 2.46 ± 0.26 |
| 10 | 4-ethylguaiacol | G | C_β_ | 2.96 ± 0.07 | 2.43 ± 0.12 | 3.16 ± 0.29 | 3.04 ± 0.55 | 2.77 ± 0.58 | 2.67 ± 0.14 | 2.65 ± 0.17 |
| 11 | 4-vinylguaiacol | G | C_β_ | 19.66 ± 0.84 | 13.42 ± 0.21 | 8.73 ± 0.50 | 10.08 ± 0.59 | 13.23 ± 0.61 | 5.16 ± 0.39 | 9.32 ± 0.53 |
| 12 | Syringol | S | 0 | 7.03 ± 0.18 | 8.14 ± 0.15 | 10.67 ± 0.33 | 12.18 ± 0.78 | 9.43 ± 0.67 | 9.95 ± 0.69 | 11.20 ± 0.65 |
| 13 | Eugenol | G | C_γ_ | 1.46 ± 0.15 | 1.82 ± 0.16 | 1.42 ± 0.34 | 1.72 ± 0.26 | 1.58 ± 0.24 | 1.95 ± 0.60 | 1.89 ± 0.39 |
| 14 | Vanillin | G | C_α_ | 1.84 ± 0.03 | 2.12 ± 0.18 | 1.83 ± 0.18 | 2.15 ± 0.62 | 2.43 ± 0.30 | 2.06 ± 0.30 | 2.04 ± 0.29 |
| 15 | Cis-isoeugenol | G | C_γ_ | 1.22 ± 0.17 | 1.04 ± 0.21 | 1.16 ± 0.20 | 1.19 ± 0.34 | 1.17 ± 0.21 | 1.08 ± 0.34 | 1.20 ± 0.19 |
| 16 | 4-methylsyringol | S | C_α_ | 1.43 ± 0.30 | 1.47 ± 0.04 | 0.78 ± 0.06 | 0.88 ± 0.09 | 1.98 ± 0.42 | 0.92 ± 0.15 | 1.00 ± 0.10 |
| 17 | Trans-isoeugenol | G | C_γ_ | 3.46 ± 0.25 | 3.23 ± 0.09 | 2.16 ± 0.04 | 2.16 ± 0.16 | 2.76 ± 0.49 | 1.34 ± 0.08 | 2.49 ± 0.16 |
| 18 | Homovanillin | G | C_β_ | 1.01 ± 0.29 | 2.16 ± 0.04 | 1.91 ± 0.08 | 2.01 ± 0.13 | 1.77 ± 0.13 | 1.63 ± 0.15 | 1.69 ± 0.16 |
| 19 | 4-ethylsyringol | S | C_β_ | 3.81 ± 0.71 | 5.67 ± 0.13 | 10.15 ± 0.56 | 7.07 ± 0.45 | 5.44 ± 1.84 | 9.95 ± 1.46 | 5.96 ± 0.33 |
| 20 | Guaiacylacetone | G | C_γ_ | 2.55 ± 0.57 | 4.11 ± 0.12 | 4.27 ± 1.20 | 6.96 ± 0.33 | 3.38 ± 0.84 | 11.21 ± 0.19 | 5.62 ± 1.68 |
| 21 | 4-vinylsyringol | S | C_β_ | 6.43 ± 0.25 | 4.94 ± 0.14 | 2.03 ± 0.18 | 2.06 ± 0.36 | 5.87 ± 0.71 | 1.40 ± 0.19 | 3.12 ± 0.10 |
| 22 | Vanillic acid methyl ester | G | C_α_ | 0 ± 0 | 1.79 ± 0.14 | 2.05 ± 0.05 | 2.19 ± 0.43 | 1.73 ± 0.67 | 2.56 ± 0.35 | 2.34 ± 0.51 |
| 23 | Vanilloyl acetaldehyde | G | C_γ_ | 0.99 ± 0.01 | 0.94 ± 0.02 | 0.44 ± 0.12 | 0.57 ± 0.10 | 1.14 ± 0.13 | 0.42 ± 0.15 | 0.90 ± 0.05 |
| 24 | Cis-4-propenylsyringol | S | C_γ_ | 1.04 ± 0.25 | 1.03 ± 0.02 | 0.67 ± 0.04 | 0.84 ± 0.09 | 1.55 ± 0.70 | 0.67 ± 0.29 | 1.13 ± 0.04 |
| 25 | Syringaldehyde | S | C_α_ | 0.94 ± 0.10 | 1.87 ± 0.13 | 0.76 ± 0.03 | 0.90 ± 0.14 | 1.91 ± 0.22 | 0.52 ± 0.08 | 1.26 ± 0.05 |
| 26 | 4-propinylsyringol | S | C_γ_ | 0.47 ± 0.10 | 0.57 ± 0.05 | 0 ± 0 | 0 ± 0 | 0.59 ± 0.05 | 0 ± 0 | 0 ± 0 |
| 27 | Trans-4-propenylsyringol | S | C_γ_ | 3.07 ± 0.26 | 2.89 ± 0.16 | 1.40 ± 0.10 | 1.52 ± 0.22 | 3.60 ± 0.46 | 0.97 ± 0.08 | 2.17 ± 0.26 |
| 28 | Homosyringaldehyde | S | C_γ_ | 1.48 ± 0.04 | 2.43 ± 0.08 | 2.20 ± 0.17 | 2.79 ± 0.37 | 2.69 ± 0.31 | 1.71 ± 0.31 | 2.40 ± 0.12 |
| 29 | Acetosyringone | S | C_β_ | 0.77 ± 0.04 | 0.55 ± 0.14 | 0 ± 0 | 0 ± 0 | 0.75 ± 0.26 | 0 ± 0 | 0 ± 0 |
| 30 | Trans-coniferyl alcohol | G | C_γ_ | 2.20 ± 0.36 | 1.23 ± 0.59 | 0 ± 0 | 0 ± 0 | 0.78 ± 0.46 | 0 ± 0 | 0 ± 0 |
| 31 | Syringylacetone | S | C_γ_ | 1.05 ± 0.22 | 1.41 ± 0.34 | 1.47 ± 0.06 | 1.88 ± 0.16 | 1.95 ± 0.16 | 1.32 ± 0.07 | 1.74 ± 0.04 |
| 32 | Propiosyringone | S | C_γ_ | 0.90 ± 0.07 | 1.17 ± 0.12 | 1.09 ± 0.04 | 1.34 ± 0.18 | 0.75 ± 0.06 | 0.95 ± 0.07 | 0.91 ± 0.05 |
| 33 | Trans-sinapaldehyde | S | C_γ_ | 0.90 ± 0.13 | 0.64 ± 0.20 | 0 ± 0 | 0 ± 0 | 1.48 ± 0.13 | 0 ± 0 | 0 ± 0 |
| 34 | Trans-sinapyl alcohol | S | C_γ_ | 0.66 ± 0.05 | 0.12 ± 0.10 | 0 ± 0 | 0 ± 0 | 0.26 ± 0.03 | 0 ± 0 | 0 ± 0 |

Values are expressed as the means determined for three replicates ± SE.

The amount of each compound is expressed as percentages.

The compositional data were calculated based on H+G+S+mis.=100%.

^a^H:lignin H units, G:lignin G units, S: lignin S units.

^b^Lignin-derived phenols with 0, 1, 2 and 3 carbons in the side chain.

^c^Miscellaneous.

^d^Two replicates.

# Table S3. The assignments of 13C-1H peaks in HSQC spectra.

| Label | δ _C_ / δ _H_ (ppm) | Assignments |
| --- | --- | --- |
| Aromatic region | | |
| *p*CA_α_ | 144.8/7.41 | C_α_-H_α_ in *p*-coumarate (*p*CA) and ferulate (FA) |
| PB_2,6_ | 131.8/7.67 | C_2_-H_2_ and C_6_-H_6_ in *p*-hydroxy-benzoate (PB) |
| *p*CA_2,6_ | 130.1/7.45 | C_2_-H_2_ and C_6_-H_6_ in p-coumarate (*p*CA) |
| H_2,6_ | 127.8/7.22 | C_2,6_-H_2,6_ in p-hydroxyphenyl units (H) |
| FA_6_ | 123.2/7.15 | C_6_-H_6_ in ferulate (FA) |
| G′_6_ | 123.2/7.33 | C_6_-H_6_ in guaiacyl units (G) |
| G_6_ | 119.0/6.78 | C_6_-H_6_ in guaiacyl units (G) |
| G_5_ | 115.1/6.92 | C_5_-H_5_ in etherified guaiacyl units (G) |
| *p*CA_3,5_ | 115.5/6.77 | C_3_-H_3_ and C_5_-H_5_ in *p*-coumarate (*p*CA) |
| G_5_ | 114.9/6.70 | C_5_-H_5_ in guaiacyl units (G) |
| *p*CA_β_ | 113.5/6.27 | C_β_-H_β_ in *p*-coumarate (*p*CA) and ferulate (FA) |
| FA_2_ | 111.0/7.32 | C_2_-H_2_ in ferulate (FA) |
| G′_2_ | 111.2/7.47 | C_2_-H_2_ in guaiacyl units (G′) |
| G_2_ | 110.9/6.99 | C_2_-H_2_ in guaiacyl units (G) |
| S′_2,6_ | 106.3/7.32 | C_2,6_-H_2,6_ in oxidized S units (S′) |
| S_2,6_ | 103.8/6.69 | C_2_-H_2_ and C_6_-H_6_ in etherified syringyl units (S) |
|  | | |
| Aliphatic (Side chain) region | | |
| B_α_ | 86.8/5.43 | C_α_-H_α_ in phenylcoumaran substructures (PB) |
| A_β_(S) | 85.9/4.10 | C_β_-H_β_ in β-O-4′substructures linked (A) to a S unit |
| D_β_ | 85.3/3.85 | C_β_-H_β_ in dibenzodioxocin substructures (D) |
| C_α_ | 84.8/4.65 | C_α_-H_α_ in β-β′ resinol substructures (C) |
| A_β_(G) | 83.4/4.27 | C_β_-H_β_ in β-O-4′substructures (A) linked to a G unit |
| D_α_ | 83.3/4.81 | C_α_-H_α_ in dibenzodioxocin substructures (D) |
| A_β_(H) | 82.9/4.48 | C_β_-H_β_ in β-O-4′substructures (A) linked to a H-unit |
| Aox_β_ | 82.7/5.22 | C_β_-H_β_ in α-oxidized β-O-4′ substructures (Aox) |
| D_α_ | 81.2/5.10 | C_α_-H_α_ in spirodienone substructures (D) |
| A′_β_(G) | 80.8/4.52 | C_β_-H_β_ in γ-acylated β-O-4′substructures linked to a G-unit (A′) |
| A_α_(S) | 71.8/4.83 | C_α_-H_α_ in β-O-4′substructures (A) linked to a S-unit |
| A_α_(G) | 70.9/4.71 | C_α_-H_α_ in β-O-4′ substructures (A) linked to a G-unit |
| C_γ_ | 71.0/3.81 and 4.17 | C_γ_-H_γ_ in β-β′ resinol substructures (C) |
| A′_γ_ | 63.5/3.83 and 4.30 | C_γ_-H_γ_ in γ-acylated β-O-4′ substructures(A′) |
| B_γ_ | 62.6/3.67 | C_γ_-H_γ_ in phenylcoumaran substructures (B) |
| D_β_ | 59.5/2.75 | C_β_-H_β_ in spirodienone substructures (D) |
| A_γ_ | 59.4/3.35 – 3.80 | C_γ_-H_γ_ in γ-hydroxylated β-O-4′substructures (A) |
| −OMe | 55.6/3.73 | C-H in methoxyls |
| C_β_ | 53.5/3.05 | C_β_-H_β_ in β-β′ resinol substructures (C) |
| B_β_ | 53.1/3.43 | C_β_-H_β_ in phenylcoumaran substructures (B) |

# Figure S1. Weight loss of poplar and wheat straw for different treatments.





# Figure S2. Py-GC/MS profiles of raw poplar A and wheat straw B.

(See Table S1 and Table S2 for peak identification and relative molar areas, the structure of the labeled peaks is shown in Figure S3.)


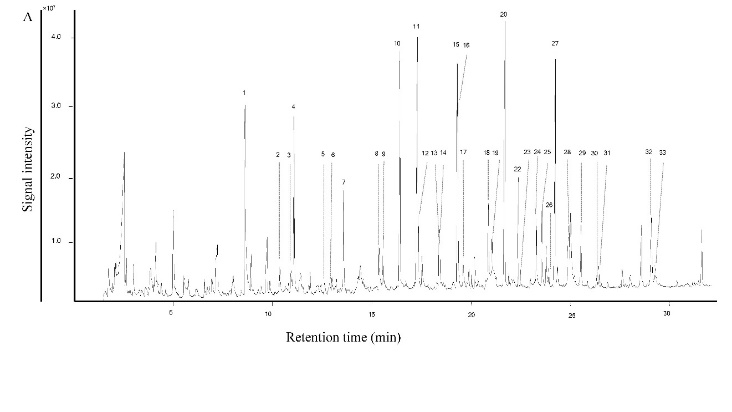


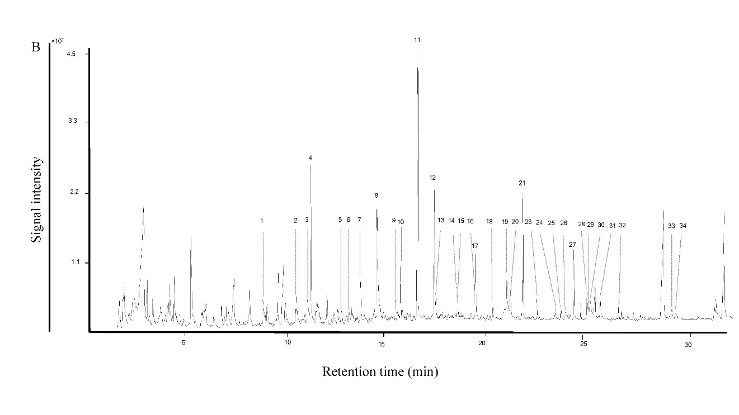


# Figure S3. Structures of the wheat straw (poplar) lignin derived compounds released from the Py-GC/MS.

Identified poplar lignin-derived subunits are shown in brackets.


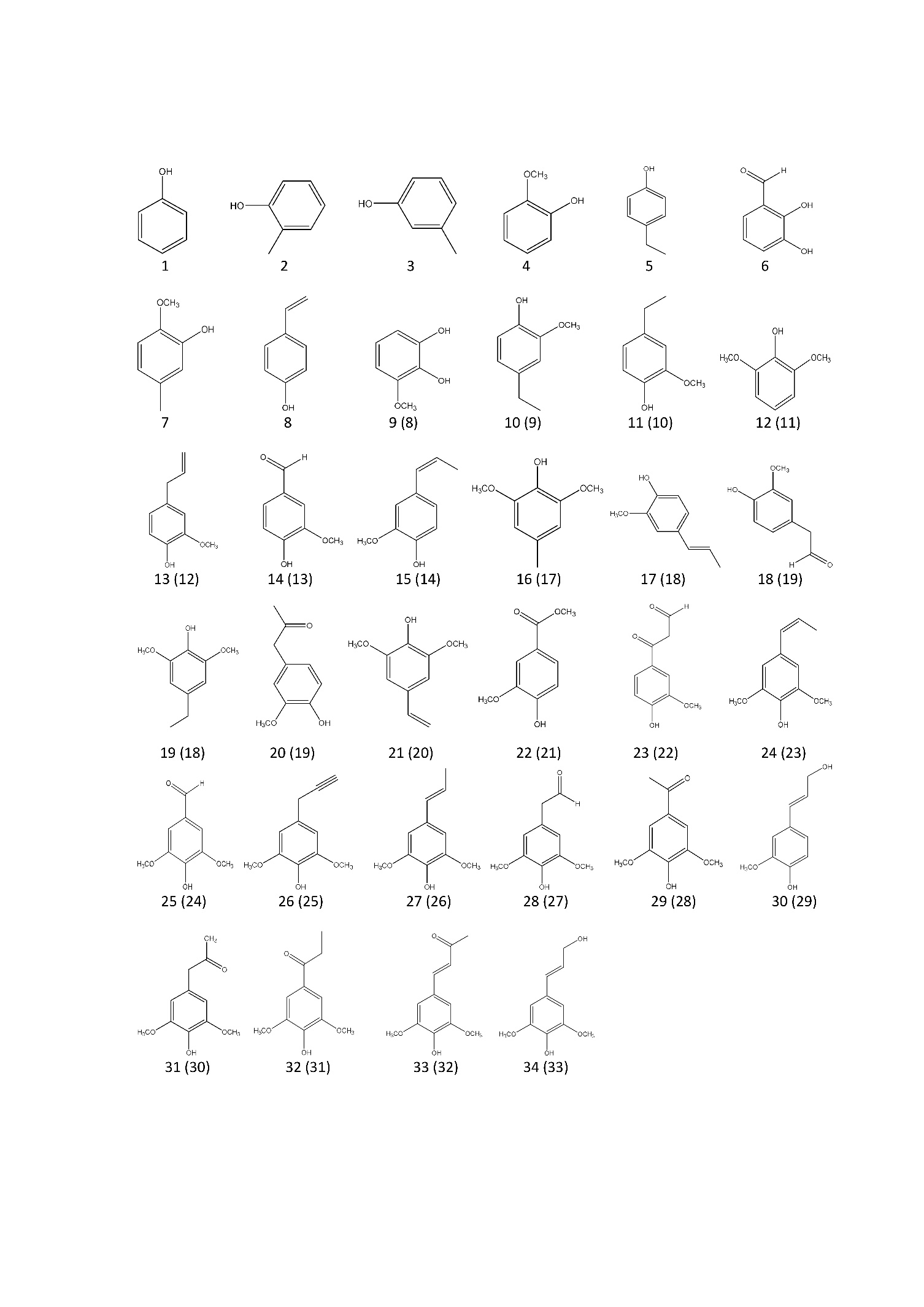


# Figure S4. 2D HSQC NMR spectra of fungal-treated poplar.

Po (a, e), Pv (b, f), PoM (c, g), PvM (d, h). a, b, c, d: aromatic (δC / δH 100-140/6.0-8.0) region, and e, f, g, h: aliphatic (δC / δH 50–90/2.5–6.0) region. The annotated substructures of correlated peaks are shown in Figure S6 with color coded. Unassigned, amino acid and carbonhydrate residues are shown in gray.


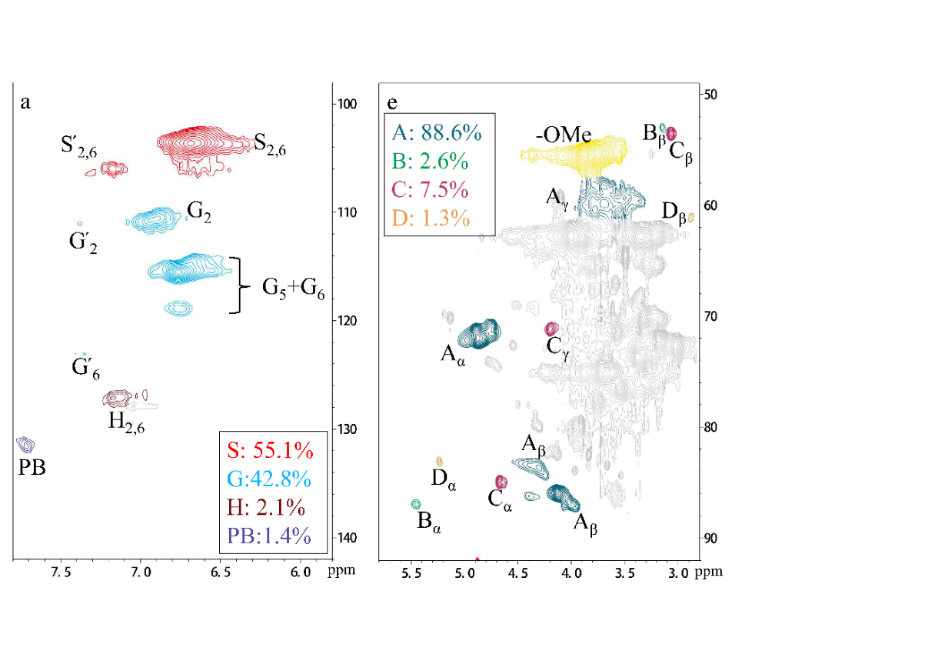


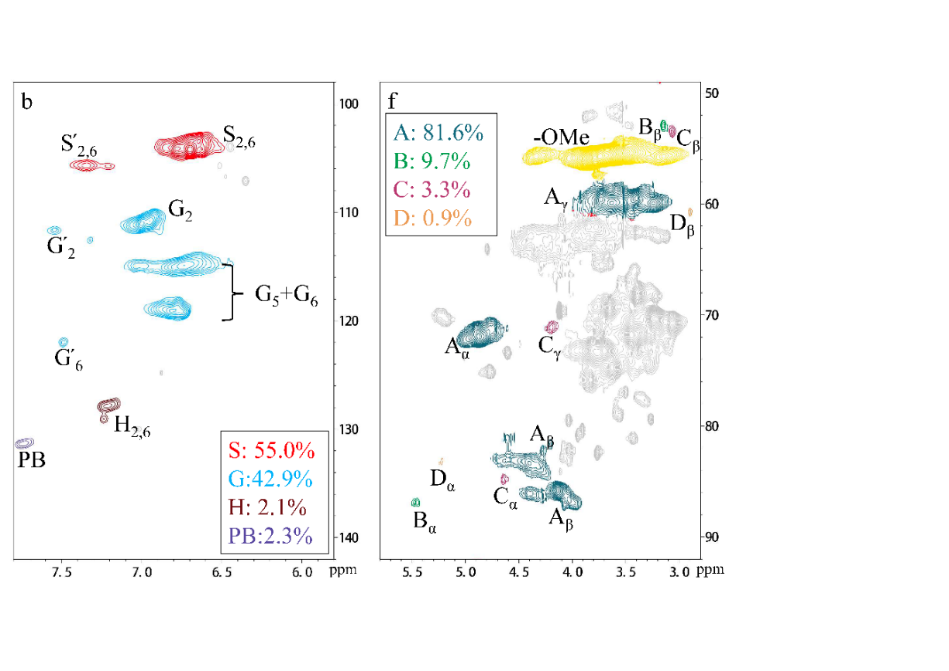


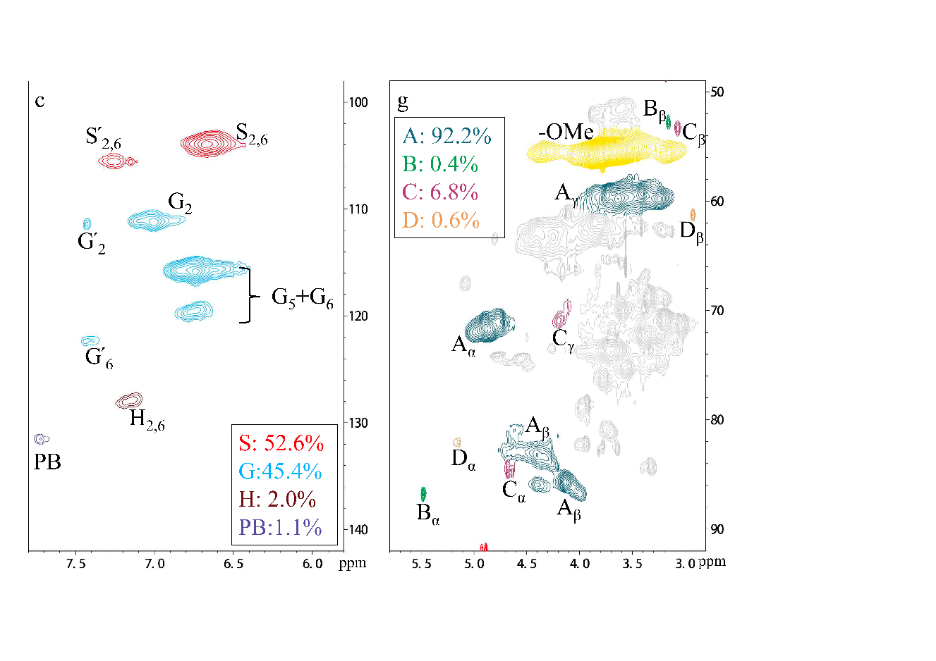


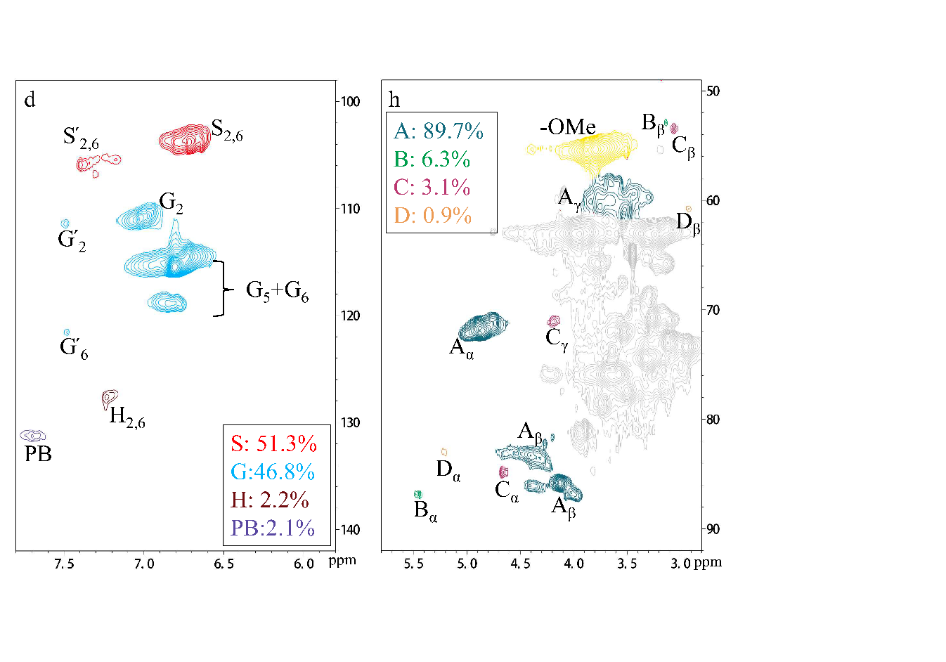


# Figure S5. 2D HSQC NMR spectra of raw and fungal-treated wheat straw.

Raw wheat straw (a, h), Pc (b, i), Po (c, j), Pv (d, k), PcM (e, l), PoM (f, m), PvM (g, n), a, b, c, d, e, f, g: aromatic (δC / δH 100-140/6.0-8.0) region, and h, j, k, l, m, n: aliphatic (δC / δH 50–90/2.5–6.0) region. The annotated substructures of correlated peaks are shown in Figure S6. Unassigned, amino acid and carbonhydrate residues are shown in gray.


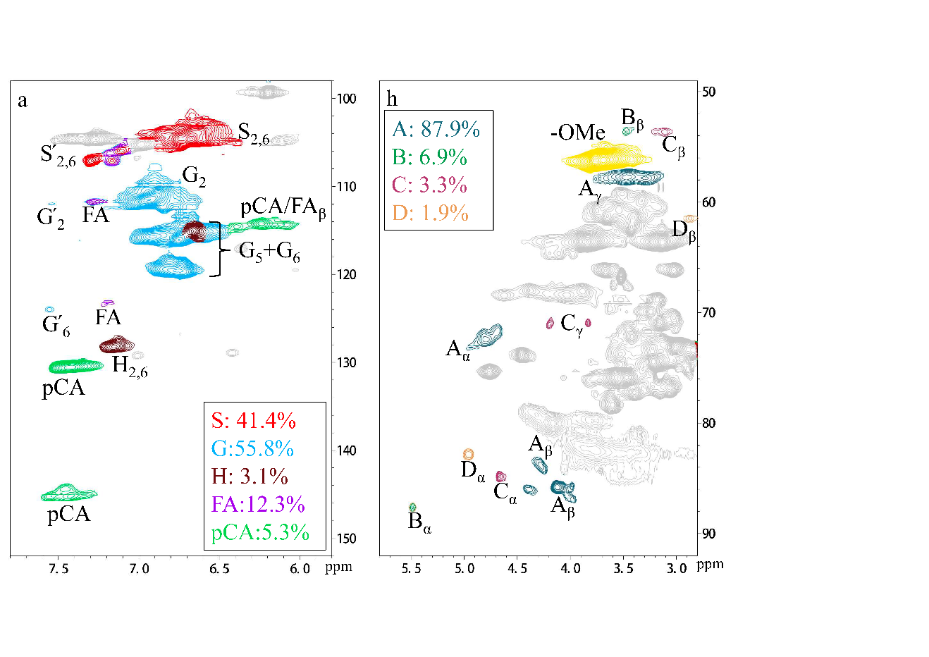


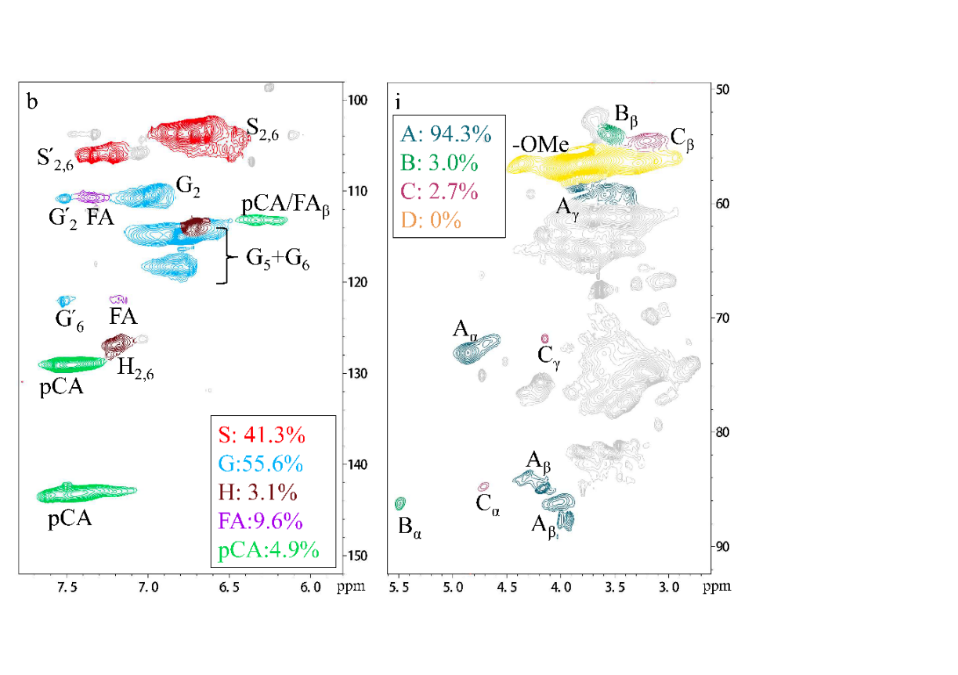


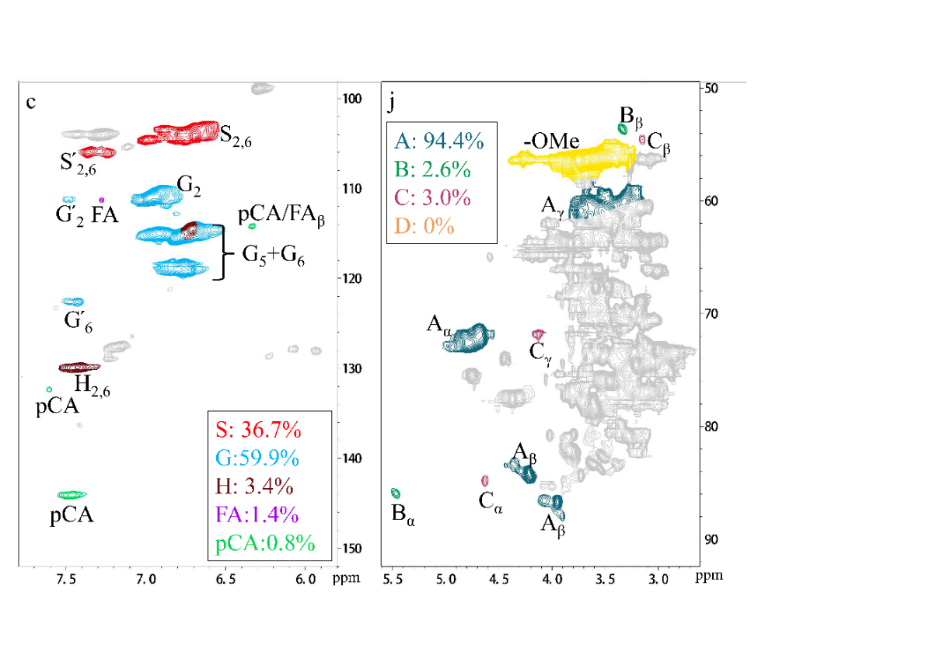


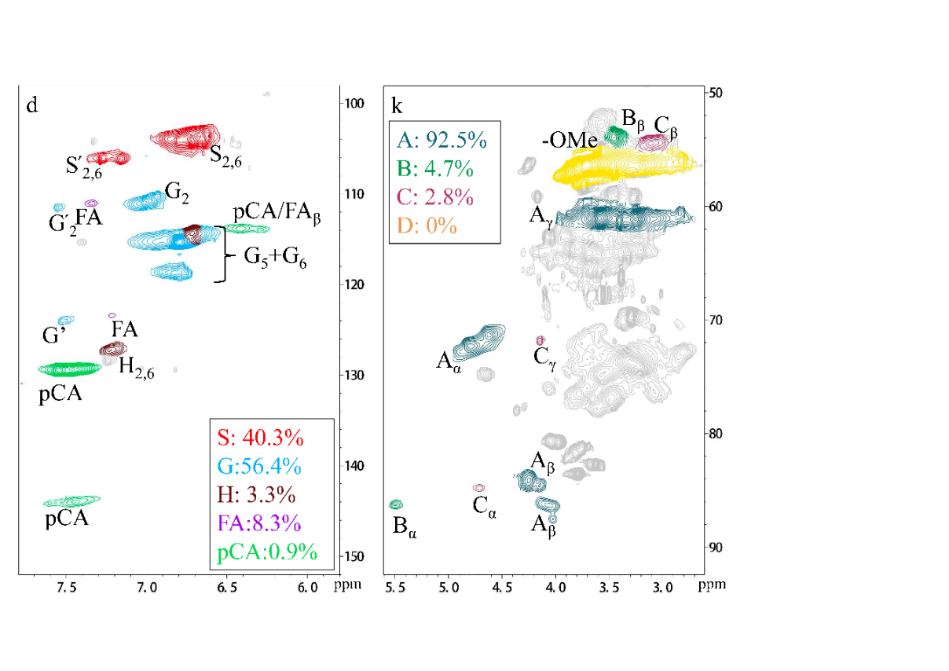


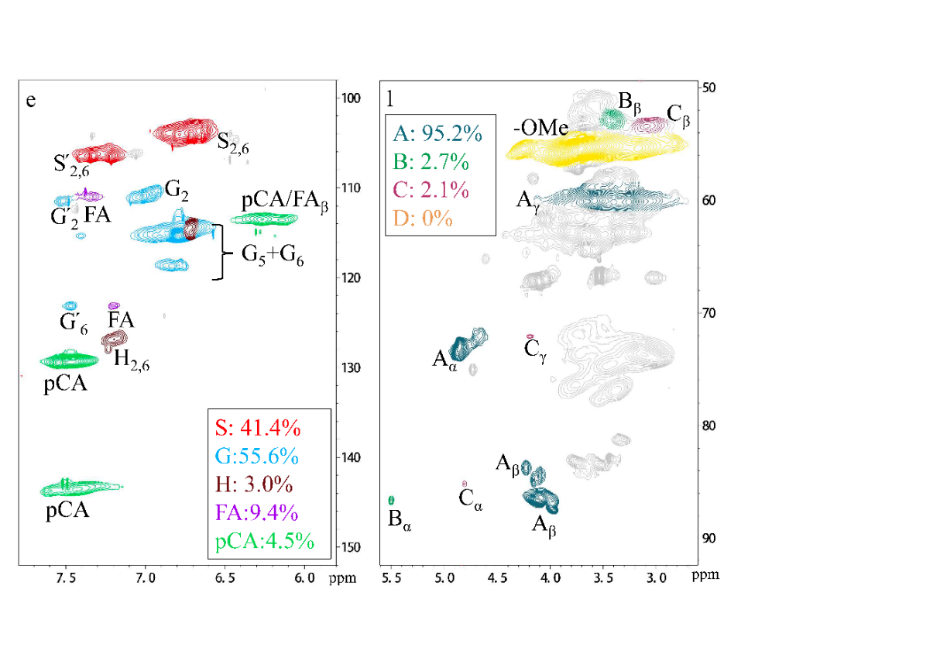


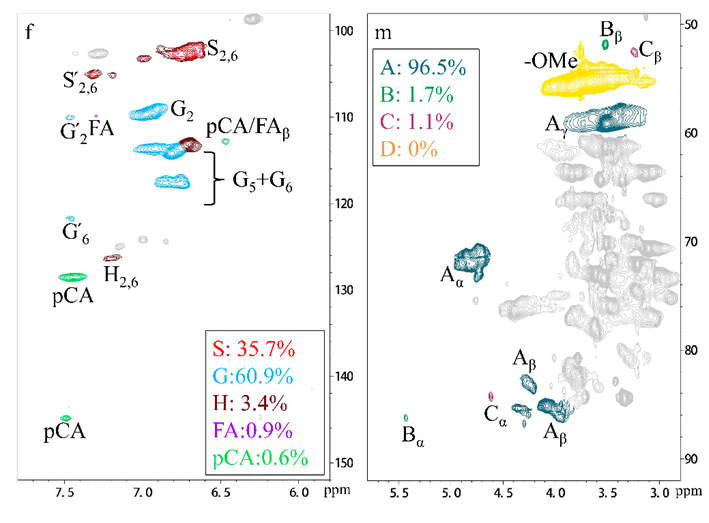


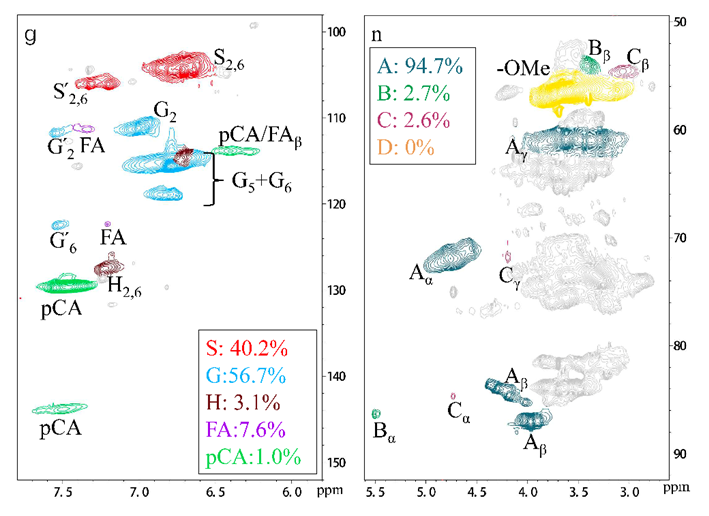


# Figure S6. HSQC NMR annotated structure.

a: relative interunit linkages (A: β-O-4; Aox: Cα-oxidized β-O-4; A՛: γ-acylated β-O-4; B: β-5; C: β-β; D: β-1). b: lignin subunits (S: syringyl unit; S՛: Cα-oxidized syringyl unit; G: guaiacyl unit; G՛: Cα-oxidized guaiacyl unit; H: p-hydroxyphenyl unit; FA: ferulate; PCA: p-coumarate; PB: p-hydroxybenzoate). OMe-OCH3. Color match the assigned peaks in Figure 4, Figure S5 and Figure S6.


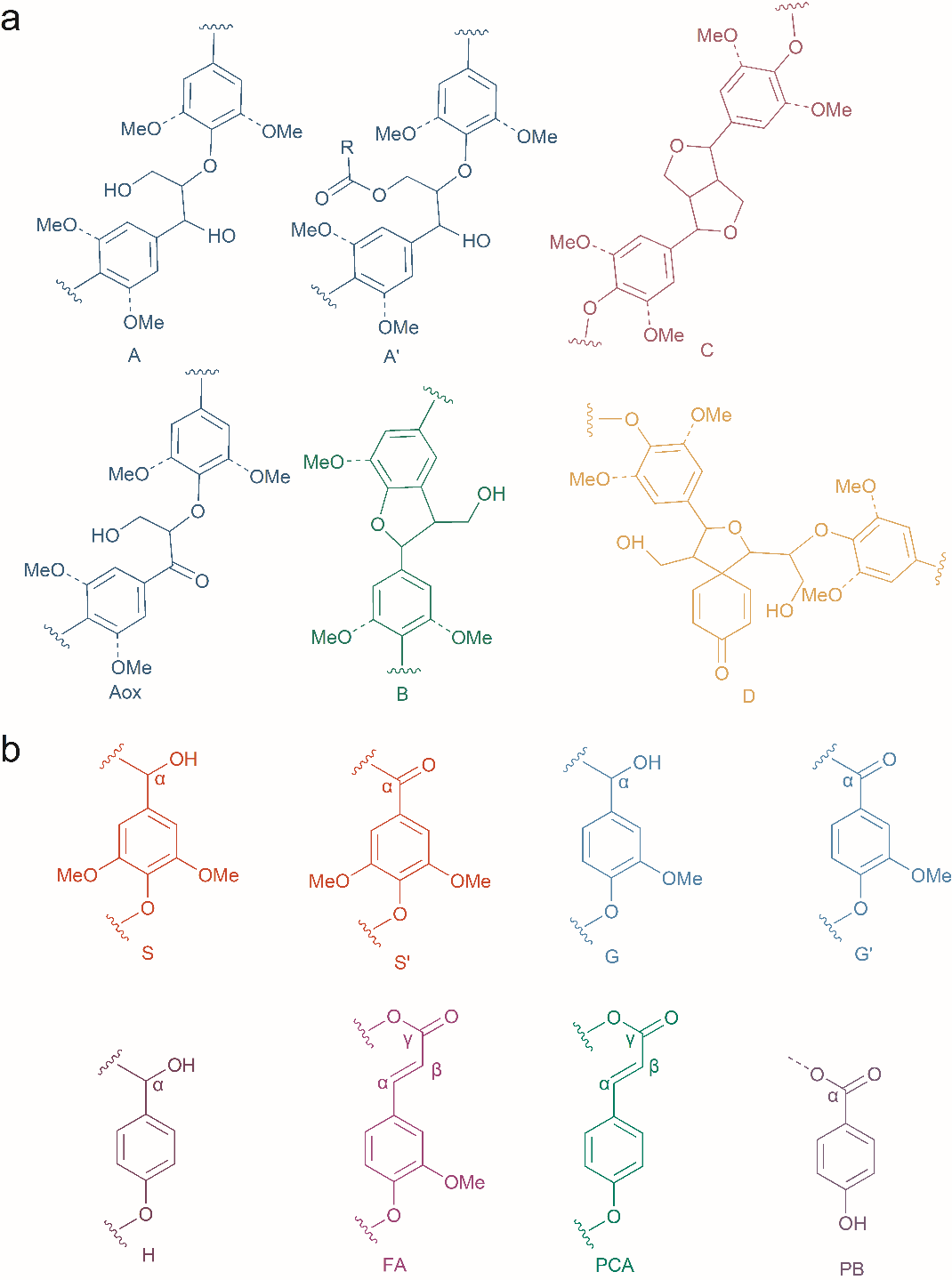

Supplement: Supplementary file 1 — Additional file 1: Table S1. Relative abundance of the phenolic compounds derived peaks identified in the Py-GC/MS of poplar. Table S2. Relative abundance of the phenolic compounds derived peaks identified in the Py-GC-MS of wheat straw.4 Table S3. The assignments of 13C-1H peaks in HSQC spectra. Figure S1. Weight loss of poplar and wheat straw for different treatments. Figure S2. Py-GC/MS profiles of raw poplar A and wheat straw B.Figure S3. Structures of the wheat straw (poplar) lignin derived compounds released from the Py-GC/MS. Figure S4. 2D HSQC NMR spectra of fungal-treated poplar. Figure S5. 2D HSQC NMR spectra of raw and fungal-treated wheat straw. Figure S6. HSQC NMR annotated structure. [file 13068_2021_2024_MOESM1_ESM.docx]
